# Artesunate directly targets glucosylceramidase to suppress hepatocellular carcinoma proliferation and trigger apoptosis

**DOI:** 10.1016/j.gendis.2026.102045

**Published:** 2026-01-20

**Authors:** Xia Mao, Xiangying Yan, Yawen Chen, Bingbing Cai, Wenjia Chen, Ya Lin, Na Lin, Yanqiong Zhang

**Affiliations:** a State Key Laboratory for Quality Ensurance and Sustainable Use of Dao-di Herbs, Institute of Chinese Materia Medica, China Academy of Chinese Medical Sciences, Beijing 100700, China; b College of Pharmacy, Fujian University of Traditional Chinese Medicine, Fuzhou, Fujian 350122, China

**Keywords:** Artesunate, Direct binding site, Glucosylceramidase, Hepatocellular carcinoma, Molecular mechanism

## Abstract

Our previous preclinical study determined artesunate as a candidate drug for hepatocellular carcinoma (HCC) and identified glucosylceramidase (GBA) as one of its direct targets. This research aimed to identify the binding sites of GBA with artesunate and the potential anti-HCC mechanisms, which remain unclear. Artesunate effectively suppressed cell viability and proliferation, and enhanced apoptosis of HCC cell lines with more sensitivity in HepG2 than MHCC-97H cells. Network calculation and a series of *in vivo* and *in vitro* experimental data demonstrated that the apoptosis-related GBA-ceramide-CTSD-BID-BAX signaling was one of the key putative target pathways by which artesunate may inhibit the malignant progression of HCC. Furthermore, through integrated computational and experimental approaches, we identified Y313, E340, and N396 as critical binding residues within the GBA active site. Mutagenesis studies revealed that these residues were indispensable for the interaction, with E340R and N396R mutations exhibiting the most pronounced impairment in binding affinity and enzymatic activity, respectively. Crucially, disrupting this binding interface abolished artesunate's ability to modulate the downstream apoptotic pathway. Our findings provide the first structural and mechanistic elucidation of artesunate's target engagement with GBA, unveiling a specific signaling cascade for its anti-HCC activity and establishing a foundational framework for developing novel GBA-targeted therapies.

## Introduction

Hepatocellular carcinoma (HCC) is a primary liver malignancy composed of epithelial cells with hepatocellular differentiation, accounting for 75%–85% of all primary liver cancer.^1^ It is the third leading cause of cancer deaths worldwide and ranks sixth in terms of incidence. According to annual projections by the World Health Organization, more than one million patients are estimated to die from HCC in 2030, seriously threatening the health and lives of the population.^2^ Treatment options for patients with HCC have been outlined in both national and international guidelines, with slight differences, and include liver transplantation, surgical resection, percutaneous ablation, and radiation, as well as trans-arterial and systemic therapies (first-line and second-line agents), based on the Barcelona Clinic Liver Cancer staging system.^3^ Despite substantial progress in these locoregional and systemic therapies, only one-third of patients with HCC respond with satisfying clinical efficacy, and the remaining patients may not respond and ultimately succumb to the disease.^4^ Additionally, the existing medication agents often exert various unfavorable chemical characteristics, such as being highly hydrophilic, orally inactive, and having poor membrane permeability, a short plasma half-life, potential severe side effects, and weak tumor-targeting properties, that seriously limit their clinical application.^5^^,^^6^ Therefore, the identification of novel molecular targets and the development of more effective, targeted therapeutics are urgently needed.

Artesunate, a US FDA-approved derivative of artemisinin, has been widely used clinically with the benefits of excellent water solubility, high bioavailability, and fine activity.^7^ In recent years, artesunate has attracted more attention for its prominent and broad anti-cancer potentials both *in vivo* and *in vitro*, the mechanism of which has been reported to be associated with its inhibitory effects in cancer cell proliferation and autophagy, as well as its activation of cancer cell apoptosis.^8^ Notably, our preclinical study previously confirmed the anti-HCC effects of artesunate based on the rat model of inflammation-induced HCC and an orthotopic mouse model, and also identified glucosylceramidase (GBA) as one of its direct targets to regulate lysosomal autophagy of HCC cells.^9^ The balance between autophagy and apoptosis modulates liver cell turnover and sustains intracellular homeostasis, while their dysregulation often occurs in HCC. Autophagy is interrelated with apoptosis in HCC, and it can either suppress or promote apoptosis for regulating the state of liver cancer cells.^10^ GBA overexpression has been reported to reduce reactive oxygen species and the ratio of cell apoptosis in liver tissues.^11^ However, the precise structural basis of the artesunate–GBA interaction and the specific downstream signaling pathway that connects this engagement to the execution of apoptosis remain entirely elusive.

To bridge this critical knowledge gap, the present study was designed to achieve two primary objectives. First, we sought to comprehensively characterize the anti-HCC effects of artesunate, assessing its impact on cell viability, proliferation, and apoptosis in distinct HCC cell lines. Second, and more importantly, we aimed to delineate the mechanistic pathway downstream of GBA inhibition. We employed an integrated strategy combining network pharmacology, computational simulations (molecular docking and mutagenesis), and a suite of experimental validations, including cellular thermal shift assay, microscale thermophoresis assay, and functional apoptosis assays, to precisely map the artesunate-binding residues on GBA and to elucidate the consequent pro-apoptotic signaling cascade. This work thereby provides a definitive link between target engagement and phenotypic outcome, offering new insights for targeted drug development.

## Material and methods

### Tissue specimens

The tissue specimens used here were obtained from an orthotopic mouse model based on our previous study. Male BALB/c nude mice (weight: 15 ± 2 g; age: 7–8 weeks) were purchased from Guangdong Medical Laboratory Animal Center, Guangzhou, China (license no: SCXK-2018-0186). The following six groups of tissue samples were established before and after the artesunate treatment (*n* = 3), which lasted six weeks: Control (sham operation), HepG2 (3 × 10^6^ HepG2 cells were injected into the right lobe of the liver), HepG2 with various doses of artesunate (low: 5 mg/kg; moderate: 10 mg/kg; high: 20 mg/kg), and HepG2 with the GBA activator LTI-291 at a concentration of 10 nM (lot no. S1024, Selleck, Shanghai, China) and a dose of 20 mg/kg of artesunate, which was used for inducing GBA expression in HepG2 cells.^9^

### Cell lines and cell culture

The HCC cell lines HepG2 and MHCC-97H, and THLE-2 cells were obtained from the American Type Culture Collection (Rockville, MD, USA). Cells were cultured in an incubator at 37 °C and 5% CO_2_ in Dulbecco's modified Eagle's medium (Gibco, California, USA) supplemented with 10% fetal bovine serum (Gibco, California, USA) and 1% penicillin G and streptomycin (HyClone, Logan, USA). All cell lines were authenticated via short tandem repeat profiling and confirmed to be mycoplasma-free.

### Cell transfection

Cells were plated on a 24-well culture plate overnight to reach 50%–60% confluence, and the transfection reagents were prepared. One wild-type gene and four mutant *GBA* genes were separately added into the pCDNA3.1 vector, and the C-terminal was fused with the 3 × FLAG tag to construct five eukaryotic expression plasmids (5′ end sequencing primer: CGCAAATGGGCGGTAGGCGTG; 3′ end sequencing primer: GGAAAGGACAGTGGGAGTG). They include the pCDNA3.1 vector (1.17 μg/μL), pCDNA3.1-GBA1 (2.88 μg/μL), pCDNA3.1-GBA1 (TYR313GLY, Y313G, 2.77 μg/μL), pCDNA3.1-GBA1 (GLU340ARG, E340R, 2.84 μg/μL), pCDNA3.1-GBA1 (ASN396ARG, N396R, 3.14 μg/μL), and pCDNA3.1-GBA1 (Y313G, E340R, N396R, 3.02 μg/μL). Lipofectamine 3000 reagent (0.75 μL), P3000 reagent (1 μL, Thermo Fisher Scientific, Waltham, MA, USA), and 250 ng of each plasmid were diluted and mixed in 50 μL of Opti-MEM medium (Thermo Fisher Scientific). Then, these reagents were mixed and incubated for 15 min, followed by their addition to the cells and incubation for 48 h according to the manufacturer's instructions. Fresh culture medium was added to the cultures 6 h post-transfection. The transfected cells were then treated with artesunate for 24 h and further analyzed by performing cellular thermal shift assay, cell apoptosis analysis, ELISA, immunofluorescence assay, and Western blotting analysis.

### Cell viability and cell proliferation assays

Cell viability and proliferation were assessed using CCK8 assays (lot no. CA1210, Solarbio, Beijing, China). For the cell viability experiment, 100 μL of a cell suspension (HepG2 and MHCC-97H cells) was inoculated onto a 96-well plate, with each well containing 3000 cells, and then incubated with artesunate (lot no. S24000, Shyuanye, Shanghai, China), C6-Cer (HY-19542, MCE), Synucleozid (HY-135902A, MCE), (0, 1.8 × 10^−5^, 1.8 × 10^−4^, 1.8 × 10^−3^, 1.8 × 10^−2^, 0.18, 1.8, 18, and 180 μM) for 24 h. HCC cell proliferation was monitored at 4, 8, 12, 20, 24, and 48 h following the treatment with artesunate and the positive drug 5-fluorouracil (lot no. F6627, Sigma–Aldrich, Missouri, USA). The IC_50_ of artesunate in HepG2 and MHCC-97H cells is 58.74 and 237.30 μM, respectively. Therefore, the dosages selected for HepG2 cells were 29.27, 58.74, and 117.47 μM, and those selected for MHCC-97H cells were 118.65, 237.30, and 474.60 μM. The 5-fluorouracil doses used in HepG2 and MHCC-97H cells were 30.00 and 400.00 μM, respectively.^12^^,^^13^ The absorbance of each well was measured at 450 nm using an automated microplate reader (Olympus, Tokyo, Japan).

### Apoptosis analysis

The effects of artesunate on HepG2 cells and on pathological sections of tissue samples from an orthotopic mouse model of apoptosis were assessed based on the number of apoptotic nuclei presented after performing terminal deoxynucleotidyl transferase-mediated dUTP nick-end labeling (TUNEL; lot no. C1088, Beyotime, Shanghai, China) and Hoechst staining (lot no. C0003, Beyotime) according to the manufacturer's instructions. The staining results were photographed using a microscope (Nikon, Tokyo, Japan), and the apoptosis rates were analyzed by two independent observers who were blinded to the experimental groups via calculating the percentage of apoptotic cells, which was determined by counting the number of cells in at least five random fields for each condition, each of which was replicated three times for each “*n*".

### Network analysis

GENEMANIA (http://genemania.org/search/) is a flexible, user-friendly web interface for investigating gene function, examining gene lists, and prioritizing genes for functional assays. It extends gene lists with functionally similar genes using genomics and proteomics data.^14^ This web interface can also be used to integrate biological networks for gene prioritization, such as co-expression networks, genetic interactions, and co-localization pathways.^15^ Here, a gene–gene interaction network was constructed to screen the GBA-related genes that may interact with GBA. These GBA-related genes were then imported into the target gene list using the Database for Annotation, Visualization, and Integrated Discovery (https://david.ncifcrf.gov/home.jsp), in which the Human species was selected.^16^ Kyoto Encyclopedia of Genes and Genomes (http://www.genome.jp/kegg/) pathway analysis^17^ and the WikiPathways biological pathway database (https://www.wikipathways.org/)^18^ were used to perform functional enrichment analysis using a *P* value less than 0.05 as the criterion.

### Reverse transcription PCR

Total RNA was extracted from HepG2, MHCC-97H, and THLE-2 cells using Trizol reagent (Edlian RN0102), quantified, and reverse-transcribed into cDNA using an appropriate amount of RNA with a reverse transcription kit (EXONGEN, A502). This cDNA served as the template for amplification. mRNA expression levels of SNCA (F: GCTGATTGGTGGAAAGGAAA; R: CACGGTCACAGGTTACAACG) and β-actin (F: TCCTCCTGAGCGCAAGTACTCC; R: CATACTCCTGCTTGCTGATCCAC) were quantified.

### Flow cytometry

Cells in logarithmic growth phase were harvested, and digested with trypsin after discarding the medium, followed by centrifugation to remove the supernatant, twice with phosphate buffer saline (PBS), resuspension in 1 mL PBS, and apoptosis detection using the Annexin V-FITC Apoptosis Detection Kit (Biyun Tian, C1062M). The experimental results were analyzed using FlowJo v9 software.

### Cellular thermal shift assay

HepG2 cells were seeded on 100-mm culture dishes until 80% confluence was reached and treated with fetal bovine serum-free medium containing 0.1% dimethyl sulfoxide (DMSO) or 29.27 μM artesunate for 6 h in a cell incubator. The cells were washed with PBS twice, detached using trypsin, and collected using medium containing 10% fetal bovine serum. Each group of cells (100 μL) was evenly added to a 0.2 mL PCR tube and heated at the indicated temperature (37, 43, 49, 55, 61, and 67 °C) for 5 min in the PCR machine. One of the tubes was aliquoted directly on ice without receiving heating treatment. Then, the tubes were immediately transferred to ice for 5 min, freeze-thawed repeatedly three times with liquid nitrogen, and centrifuged at 12,000 *g* and 4 °C for 15 min to obtain the supernatant. The cell supernatant was collected for protein denaturation, and the GBA expression level was analyzed using Western blotting.

### Surface plasmon resonance

Surface plasmon resonance detection was conducted to confirm the binding affinities of GBA protein with artesunate and GBA inhibitor conduritol B epoxide (CBE) using a Biacore 8K instrument (BIACORE 8K, GE, Sweden), and Biacore 8K evaluation software 2.0 (GE Healthcare) was used for calculating related K_D_ values. Detailed information on the protocols of surface plasmon resonance detection is provided in the supplementary materials.

### ELISA

Total proteins were extracted from the liver tissues of the orthotopic mouse model and HepG2 cells and subjected to quantitative analyses of the GBA, ceramide, caspase-3, caspase-7, caspase-8, and caspase-9 levels using ELISA kits according to the manufacturer's instructions. The ELISA kits used to detect ceremide (lot no. ml037499, ml037872-2), caspase-3 (lot no. YJ058512, ml057650-2), caspase-7 (lot no. YJ358519, ml060357-2), caspase-8 (lot no. YJ058513, ml058110-2), and caspase-9 (lot no. YJ058516, ml058111-2) were purchased from Shanghai Elisa Biotechnology Co., Ltd, Shanghai, China.

### Immunohistochemistry and immunofluorescence staining

The liver tissues were collected, fixed in 4% paraformaldehyde, and then embedded in paraffin. Paraffin sections with a thickness of 4 μm were prepared for immunohistochemistry analysis. A DAB color development kit (lot no. G1212-200, Servicebio, Wuhan, China) and a polymeric HRP detection system (lot no. PV9001, ZSGB-BIO, Beijing, China) were used. Mouse liver tissue sections were incubated with the primary antibody against α-synuclein (α-syn; 1:100, lot no. 10842-1-AP, Proteintech Group, Inc., Wuhan, China) for 2 h at 37 °C following the routine pre-processing, and then with an avidin-conjugated secondary antibody for 40 min at room temperature. After incubation with 75 μL of a DAB color-substrate solution (reagent Ⅰ:Ⅱ = 50:1) and hematoxylin, the stained sections were digitally observed using an Olympus BX50 microscope (Olympus). The immunohistochemistry score was used to evaluate the expression of the target protein α-syn.

HepG2 cells that had received different treatments were seeded on 15-mm-diameter glass coverslips, fixed in 4% paraformaldehyde at room temperature for 10 min, washed three times in PBS, and permeabilized in PBS containing 0.5% Triton X-100 (lot no. T8200, Solarbio) for 5 min. Then, they were briefly blocked with a 1% bovine serum albumin solution diluted in PBS for 10–15 min. Next, the cells were incubated with the primary antibody against α-syn (1:100, lot no. 10842-1-AP, Proteintech Group Inc.) at 4 °C overnight, followed by the corresponding fluorescently labeled secondary antibodies for 1 h. Nuclei were stained with 4′,6-diamino-2-phenylindole (DAPI). Images of the cells in randomly selected 200 × microscopic fields were captured using a Nikon ECLIPSE Ts2 fluorescence microscope (Nikon). Image analysis was carried out by two independent observers who were blinded to the experimental groups.

### Western blotting analysis

To investigate the regulatory effects of artesunate on proteins effective against HCC in the liver tissues of an orthotopic mouse model and HepG2 cells, Western blotting was performed according to standard protocols.^19^ Caspase-3, -7, -8, -9, cathepsin D (CTSD), BH3-interacting domain death agonist (BID), BCL2-associated X (BAX), B-cell lymphoma 2 (BCL-2), and GBA proteins were detected, and their detailed information is listed in Table S1. The density values of all target bands were first normalized against the internal control (β-actin) density values before being compared with the control group.

### Molecular docking simulation

Molecular docking-based virtual screening was performed using three free software packages available to academic users: AutoDock Vina (Scripps Research Institute, version 3.9.1), LeDock (Scripps Research Institute, version 9.2.0), and Schrödinger (https://www.schrodinger.com/, version 19.1.0). The Mol 2 file or Protein Data Bank (PDB) file for artesunate was downloaded from the PubChem database (https://pubchem.ncbi.nlm.nih.gov/). Eight crystal structures of GBA and their corresponding ligands were obtained from the PDB database (https://www.rcsb.org/) and used as receptors in the docking process: 1Y7V, 6Q6N, 6Q6L, 6MOZ, 2V3D, 2XWE, 2XWD, and 6Q6K. The active pockets of the eight GBA complexes were predicted using DeepSite (https://www.deepsite.ai/). The docking site of artesunate with GBA was determined based on the position of the co-crystallized ligand in the compound–protein structure.

Docking was conducted following the pre-processing of large molecules (water, solvent, and ligand removal, and hydrogenation) and small molecules using Vina. This software uses a Lamarckian Genetic Algorithm, and the compounds are sorted using a program based on the binding free energy (the best hit of 25 runs). The grid for all proteins is manually adjusted within the key binding amino acid residues, with the active site being defined as a cubic cell with 5 Å all around. Protein–ligand interactions are classified using Discovery Studio (www.ebi.ac.uk/pdbsum), and a low ligand binding energy with a receptor indicates high affinity for the target receptor, offering multicore capability, high performance, and enhanced accuracy.^20^ To use LeDock, protein preparation was carried out using the LePro module (http://www.lephar.com/) with all parameters set to default values, and the ligands required no processing. The site and dimension of the grid box were determined by referring to the positive ligands in the crystallographic complex, which was also set as a rectangular box with a radius of 5 Å. Redundancy was reduced by performing clustering using a root-mean-square deviation (RMSD) of 1 Å, and the binding pose parameter was set to 20 runs. LeDock calculations were then performed and demonstrated high accuracy and speed.^21^ Maestro, a tool implemented in the Schrödinger software suite, was used. Proteins were prepared by removing the solvent, adding hydrogen, and minimizing the presence of bound ligands, using the Protein Preparation Wizard. The other parameters were maintained at default values. LigPrep was used to generate the artesunate stereoisomers and tautomers. Grids were generated with bound co-crystallized ligands that covered the main amino acids interacting with the receptor, and the standard ligand for GBA was redocked at the catalytic site of the protein to validate the docking parameters. The RMSD between the co–crystal and redocked pose was 0.225 Å. The extra-precision (XP) mode of the Glide software was used for detailed visualization and comparison of the docked sites of the target proteins and ligands.^22^ The ligand was considered to have a strong binding affinity with the receptor if the absolute value of the docking score was higher than six.

### Alanine scanning and saturation mutations

Alanine scanning mutagenesis is a method of systematic alanine substitution that is used for identifying functional residues. Here, we further investigated the protein–ligand interaction relationship and determined the optimal alanine mutation combination using Discovery Studio software (http://www.discoverystudio.net/). Briefly, Discovery Studio Client and DS CHARMm were used to preprocess the structure of the proteins and apply the CHARMm force field. Interforce-based virtual amino acid mutation of protein–ligand complexes was performed using the “Calculate Mutation Energy (Binding)" function to screen the key amino acids at the active site and amino acid mutation targets that may reduce the binding affinity. Then, protein–ligand complexes of artesunate-6Q6N, -6Q6L, -6MOZ, -2V3D, -2XWE, and -6Q6K were further analyzed through single-point mutations to alanine (alanine scanning) and by mutating all the remaining 19 standard amino acids (saturation mutation). The mutation range included amino acids within 3 Å around the ligands. A mutation energy between −0.5 and +0.5 kcal/mol implies that it is neutral, that is, the mutation is not favorable for the binding of the substrate and residue. A mutation energy above 0.5 kcal/mol implies that it is destabilizing, indicating that it reduces the affinity and leads to a weakened interaction, whereas a mutation energy below 0.5 kcal/mol implies that it is stabilizing, suggesting that it increases the affinity and strengthens the interaction.

### Microscale thermophoresis

To confirm the specific binding sites of GBA on which artesunate acts, 293T cells were transfected with expression plasmids to induce the expression of EGFP-tagged wild-type GBA1 and mutant-type GBA1 (Y313G, E340R, N396R, and Y313G/E340R/N396R). Detailed information on the gene and primer sequences is listed in Table S2. After stable expression of the plasmids, cell lysates were collected from the 293T cells. The lysates were diluted in PBS-T buffer containing 0.05% (v/v) Tween 20 to a final concentration at which the fluorescent GFP signals were within the detection limit of the Monolith NT.115 instrument (NanoTemper Technologies, Munich, Germany). Artesunate (20 mM) was serially diluted at a 1:1 ratio to obtain 16 gradient concentrations. Artesunate-wild-type GBA1 and artesunate-mutant-type GBA1 binding reaction systems were incubated at room temperature for 30 min and then loaded into the NanoTemper glass capillaries. Micro thermophoresis was carried out using a light-emitting diode power of 80% and 80% microscale thermophoresis. The resulting dose–response curves were fitted to a one-site binding model to extract the equilibrium dissociation constant (K_d_) value using MO Affinity Analysis software at 37 °C.

### Statistical analyses

GraphPad Prism (version 8.0.1, San Diego, CA, USA) software was used to calculate and analyze the experimental data. Unpaired Student's *t-*test was used for comparisons between two groups, and one-way analysis of variance (ANOVA) with Tukey's test was used for multiple comparisons. All experiments were performed in triplicate. Experimental data acquired from this study were expressed as mean ± standard deviation from at least three independent *in vitro* experiments or from six animals in the *in vivo* experiments. Differences were considered statistically significant when the *P* value was less than 0.05.

## Results

### Artesunate potently suppresses HCC cell lines' viability and proliferation, triggering apoptosis

**Figure 1:**
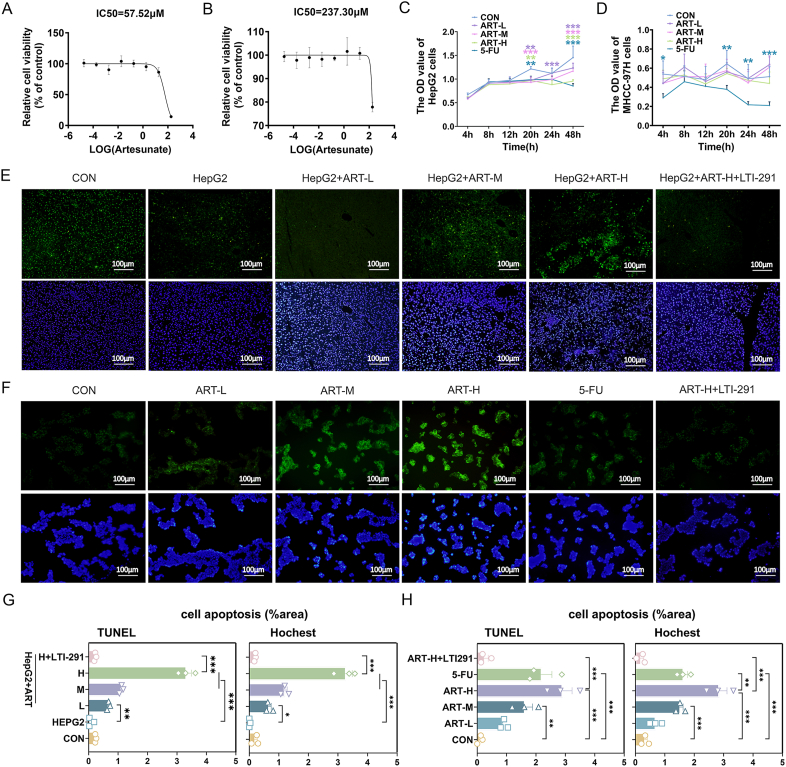
Effects of artesunate on the cell viability and proliferation of HCC cell lines (HepG2 and MHCC-97H), and its roles in the cell apoptosis based on HepG2 cells and samples obtained from an orthotopic mouse model (*n* = 3). **(A, B)** Viability inhibitory curves of HepG2 and MHCC-97H cells induced by artesunate using the CCK8 assay and the corresponding IC50 calculation. **(C, D)** Effects of artesunate on the cell proliferation of HepG2 and MHCC-97H cells using CCK8 assay. **(E, G)** Effects of artesunate on the cell apoptosis of samples obtained from the HCC orthotopic mouse model examined by TUNEL and Hoechst staining. Scale bars, 100 μm. CON: sham operation group; HepG2: 3 × 10^6^ HepG2 cells were injected into the right lobe of the liver to induce HCC orthotopic mouse model; ART-L, ART-M, ART-H: low- (5 mg/kg), middle- (10 mg/kg), high- (20 mg/kg) dose of artesunate treatment groups; ART-H + LTI-291: high dose of artesunate and GBA enzyme activator LTI-291 combined treatment group. **(F, H)** Effects of artesunate on the cell apoptosis of HepG2 cells examined by TUNEL and Hoechst staining. Scale bars, 100 μm. CON: HepG2 control group. ART-L, ART-M, ART-H: low-, middle-, and high-dose of artesunate treatment groups. 5-FU: positive drug 5-fluorouracil treatment group. ART-H + LTI-291: high dose of artesunate and GBA enzyme activator LTI-291 treatment group. The percentage of apoptotic cells derived from at least five random fields of view (*n* = 3) was analyzed by two independent observers who were blinded to the experimental groups. Data were expressed as mean ± standard deviation. *∗P* < 0.05, ∗∗*P* < 0.01, and ∗∗∗*P* < 0.001.

The cytotoxicity of artesunate in HCC cell lines (HepG2 and MHCC-97H) was assessed using a CCK8 assay. Following the treatment with artesunate at different concentrations (1.8 × 10^−5^–180 μM) for 24 h, we observed that the cytotoxicity of artesunate was stronger in HepG2 cells (IC50 value = 57.52 μM) than in MHCC-97H cells (IC50 value = 237.30 μM) (Fig. 1A and B). In addition, artesunate effectively inhibited the propagation of HepG2 cells in a dose-dependent manner 20 h after the drug treatment, whereas artesunate did not inhibit the proliferation of MHCC-97H cells, indicating that HepG2 cells may be more sensitive to the artesunate treatment than MHCC-97H cells (Fig. 1C and D). To explore the potential underlying mechanism via which artesunate suppresses cell proliferation, we performed TUNEL and Hoechst staining of hepatic tissue specimens obtained from an orthotopic mouse model and HepG2 cells treated with artesunate. As shown in Figure 1E, the TUNEL/Hoechst-negative cells exhibited well-distributed green and blue staining, and the TUNEL/Hoechst-positive cells exhibited highly condensed, brightly green and blue staining. Compared with the untreated group, low-, medium-, and high-dose artesunate clearly increased the number of TUNEL/Hoechst-positive cells dose-dependently (Fig. 1E–G). HepG2 cells treated with medium- and high-dose artesunate exhibited higher condensed chromatin enrichment in the nucleus and a strikingly higher fraction of apoptotic cells than the untreated group (Fig. 1F–H). Our previous study identified GBA as a direct target of artesunate for HCC therapy.^9^ Accordingly, the current data also demonstrated that the pro-apoptotic effect of artesunate was significantly reversed by co-treatment with the GBA activator LTI-291, providing the first clue that GBA function may be critically involved in artesunate-induced apoptosis (Fig. 1E–H).

### Artesunate directly binds to and inhibits GBA with high affinity

**Figure 2:**
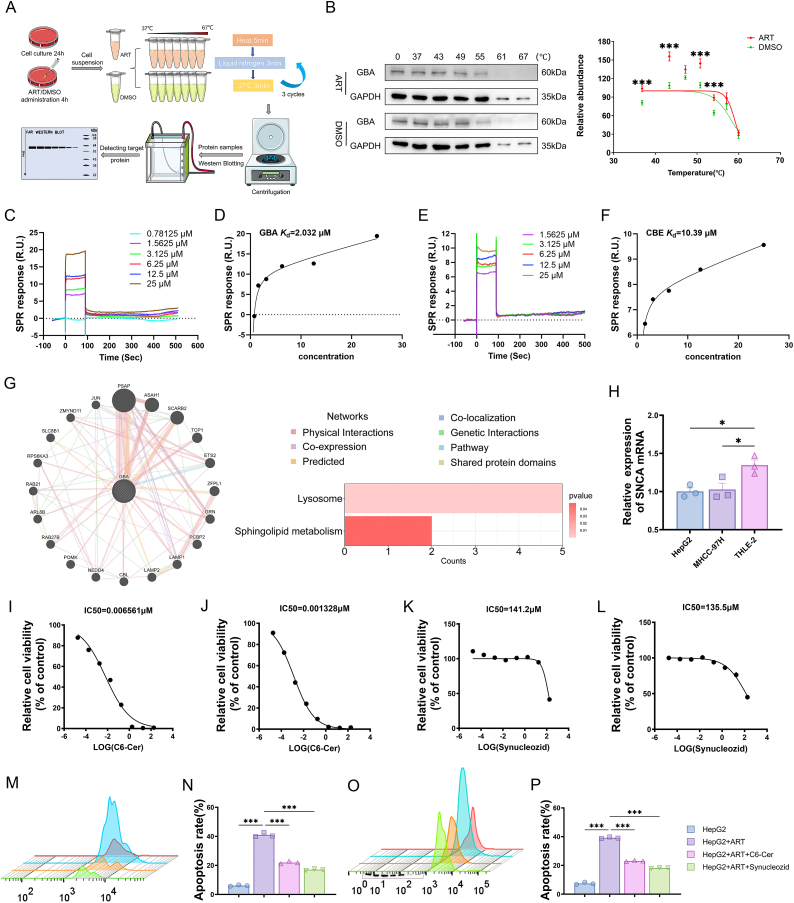
Network analysis reveals that the GBA-Ceramide-CTSD-BID-BAX signaling pathway may be a key putative target pathway of artesunate against HCC, and cellular thermal shift assay (CETSA)-Western blotting demonstrates that artesunate may bind to GBA directly. **(A)** Scheme of the CETSA-Western blotting experiment. **(B)** CETSA-Western blotting experiment confirmed direct interaction between artesunate and GBA. **(C, D)** The binding affinity between artesunate and the GBA protein was assessed by surface plasmon resonance (SPR). **(E, F)** The binding affinity between artesunate and the CBE protein was determined by SPR. **(G)** A gene–gene interaction network of GBA and its neighboring genes that may be associated with the anti-HCC effects of artesunate using GeneMANIA database, and the functional enrichment analysis of potential targets of artesunate against HCC using DAVID based on KEGG and WIKIPATHWAYS databases. **(H)** The expression of the *SNCA* gene was analyzed by reverse transcription PCR in two HCC cell lines (HepG2 and MHCC-97H) and a normal human hepatocyte line (THLE-2). **(I, J)** Viability inhibitory curves of HepG2 (I) and MHCC-97H (J) cells induced by C6-Cer using the CCK8 assay and the corresponding IC50 calculation. **(K, L)** Viability inhibitory curves of HepG2 (K) and MHCC-97H (L) cells induced by Synucleozid using the CCK8 assay and the corresponding IC50 calculation. **(M**–**P)** Flow cytometry was performed to assess the effects of C6-Cer (a lysosomal ceramide) and the α-syn inhibitor (Synucleozid) on the efficacy of artesunate. Data were expressed as mean ± standard deviation. *∗P* < 0.05, ∗∗*P* < 0.01, and ∗∗∗*P* < 0.001 versus the DMSO group.

The aforementioned finding with LTI-291 prompted us to investigate whether GBA was a direct molecular target of artesunate. To this end, we employed the cell lysate cellular thermal shift assay-Western blotting experiments. Protein extracts from HepG2 cells were treated with 29.27 μM artesunate or DMSO, and subjected to the heat pulse of cellular thermal shift assay, and soluble protein extraction and quantification (Fig. 2A). Notably, GBA displayed thermal stabilization with the interaction of artesunate. In detail, the overexpression of GBA protein was found in the artesunate-treated group compared with the DMSO-treated group, especially at 49 °C and 55 °C. Moreover, the median inhibition temperature (Tm 50) of the binding of artesunate and GBA was 65.28 °C, whereas that of DMSO and GBA was 63.76 °C (Fig. 2B). The hot melting curve shifted to the right after the artesunate treatment, indicating the direct binding of artesunate to GBA. Furthermore, we compared artesunate with the known GBA inhibitor CBE and found that artesunate exhibited stronger binding affinity (K_d_ = 2.032 μM) compared with CBE (K_d_ = 10.39 μM) (Fig. 2C–F). Collectively, these data provide evidence that artesunate is a direct and high-affinity binder of GBA.

### Inhibition of GBA by artesunate triggers a pro-apoptotic cascade via ceramide and α-synuclein

Having confirmed GBA as a direct target, we next sought to delineate the downstream apoptotic pathway. We began with a bioinformatic approach to predict GBA-related functions. GeneMANIA network analysis revealed that GBA may interact with 21 genes functionally enriched in lysosomal and sphingolipid metabolism pathways (*P* < 0.05) (Fig. 2G). GBA is a lysosomal enzyme that degrades glucosylceramide to glucose and ceramide,^23^ which is a potent proapoptotic secondary messenger lipid that transmits death-inducing signals.^24^ It specifically binds to CTSD to activate its autocatalytic function.^25^ CTSD correctly targets lysosomes, matures to an enzymatically active protease, and promotes the clearance of α-syn,^26^ which up-regulates the expression of the proapoptotic protein BAX, increases caspase activity and BID expression, and subsequently releases cytochrome C from mitochondria, making cells more susceptible to apoptosis.^27^ Based on literature and our network analysis, we proposed a working model: GBA hydrolyzes glucosylceramide to generate ceramide, which can activate CTSD to promote the clearance of α-syn; conversely, α-syn accumulation has been reported to up-regulate pro-apoptotic proteins like BAX and BID, facilitating mitochondrial apoptosis. Thus, we hypothesized that artesunate might induce apoptosis via a GBA-ceramide-CTSD-α-syn-BID-BAX axis.

To validate the relevance of key components in this model within our experimental system, we first confirmed the expression of SNCA mRNA, which encodes α-syn, in both HepG2 and MHCC-97H HCC cell lines, albeit at levels slightly lower than in normal hepatocytes (THLE-2) (Fig. 2H). This confirmed that α-syn is present and could functionally participate in our HCC models.

A critical and complex node in our hypothesis is ceramide, which literature indicates can play dual roles in cancer. To functionally dissect the roles of ceramide and α-syn in artesunate-induced apoptosis, we performed a series of rescue experiments. We first determined the non-cytotoxic concentrations of C6-Ceramide (a lysosomal-permeable ceramide) and Synucleozid (an α-syn inhibitor) for subsequent use (Fig. 2I–L). Strikingly, flow cytometry analysis revealed that co-treatment with either C6-Cer or Synucleozid significantly attenuated the apoptosis induced by artesunate in both HCC cell lines (Fig. 2M−P). These rescue experiment results lead us to two key interpretations: First, supplementing lysosomal ceramide can counteract artesunate's pro-apoptotic effect, suggesting that the depletion of ceramide following GBA inhibition is a critical pro-apoptotic signal in this context. Second, directly inhibiting α-syn also rescues the cells, confirming the pro-apoptotic role of α-syn accumulation. Therefore, the data suggest that artesunate-induced GBA inhibition coordinately regulates two pivotal events: a reduction in lysosomal ceramide and an accumulation of α-syn, which collectively drive the cell toward apoptosis.

### The GBA-mediated apoptotic pathway is robustly regulated by artesunate *in vivo* and *in vitro*

To comprehensively verify the regulatory effect of artesunate on this GBA-mediated pathway from a holistic perspective, we conducted experiments both in an orthotopic HCC mouse model and *in vitro* cell lines.

**Figure 3:**
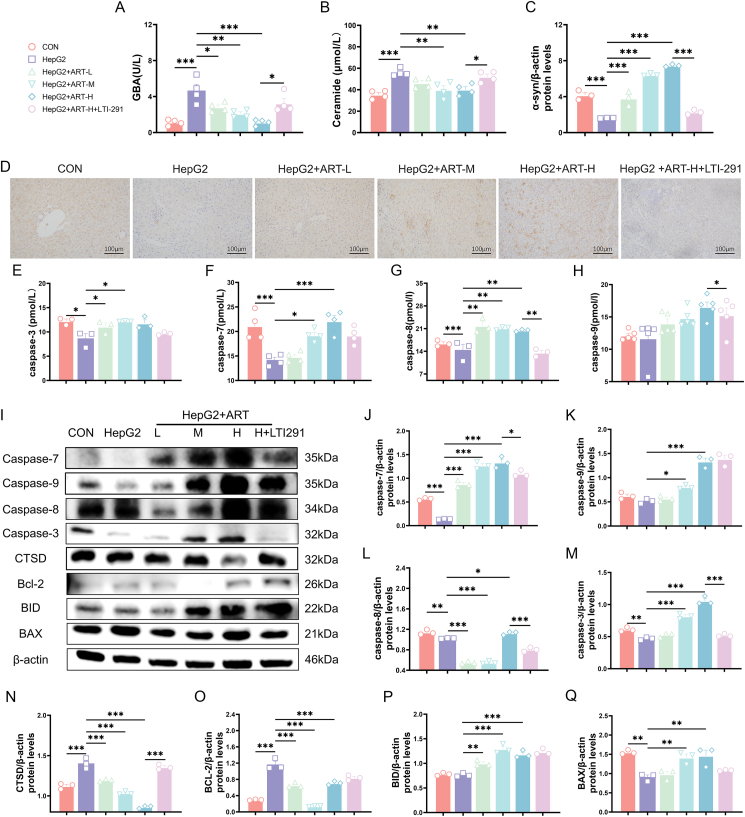
Regulatory effects of artesunate on GBA-Ceramide-CTSD-BID-BAX signaling in HCC samples obtained from an orthotopic mouse model (*n* = 3). **(A)** GBA (ELISA). **(B)** Ceramide (ELISA). **(C, D)** α-syn (IHC). Scale bars, 100 μm. **(E**–**H)** Caspase-3, Caspase-7, Caspase-8, and Caspase-9 (ELISA). **(I**–**Q)** Western blotting analysis. (I) Western blots. (J) Caspase-7. (K) Caspase-9. (L) Caspase-8. (M) Caspase-3. (N) CTSD. (O) BCL-2. (P) BID. (Q) BAX. CON: sham operation group; HepG2: 3 × 10^6^ HepG2 cells were injected into the right lobe of the liver to induce HCC orthotopic mouse model; ART-L, ART-M, ART-H: low- (5 mg/kg), middle- (10 mg/kg), high- (20 mg/kg) dose of artesunate treatment groups; ART-H + LTI-291: high dose of artesunate and GBA enzyme activator LTI-291 combined treatment group. Data were expressed as mean ± standard deviation. *∗P* < 0.05, ∗∗*P* < 0.01, and ∗∗∗*P* < 0.001.

In the *in vivo* model, we found that the activities of GBA and ceramide were markedly higher in the HepG2 group than in the normal control group, and were partially suppressed by the treatment with medium and high doses of artesunate. The regulatory effects of high-dose artesunate on the activities of both GBA and ceramide were impaired after the combined treatment with LTI-291 (Fig. 3A and B). Immunohistochemistry analysis revealed a decrease in the number of α-syn-positive cells in the HepG2 group compared with that in the control group, which was significantly reversed by the treatment with artesunate in a dose-dependent manner, and antagonized after LTI-291 was added (Fig. 3C and D). As the key putative target pathway was involved in cell apoptosis, the regulatory effects of artesunate on the enzymatic activities and expression levels of various apoptosis promoters, such as caspase-3, caspase-7, caspase-8, and caspase-9, as well as the protein expression levels of CTSD and apoptotic markers (BID, BAX, and BCL-2), were explored using ELISA and Western blotting analysis. The results showed that artesunate effectively enhanced the enzymatic activities of caspase-3, caspase-7, caspase-8, and caspase-9 in the HepG2 group (Fig. 3E–H), consistent with the protein expression levels detected via Western blotting analysis (Fig. 3I, J–M). The expression of both CTSD and BCL-2 was significantly higher in the HepG2 group than in the control group, and the expression of BAX and BID tended to be lower in the HepG2 group than in the control group. In contrast, the treatment with artesunate significantly reversed the dysregulation of the aforementioned proteins, whose functions were all weakened by the combined treatment with LTI-291 (Fig. 3I, N–Q).

**Figure 4:**
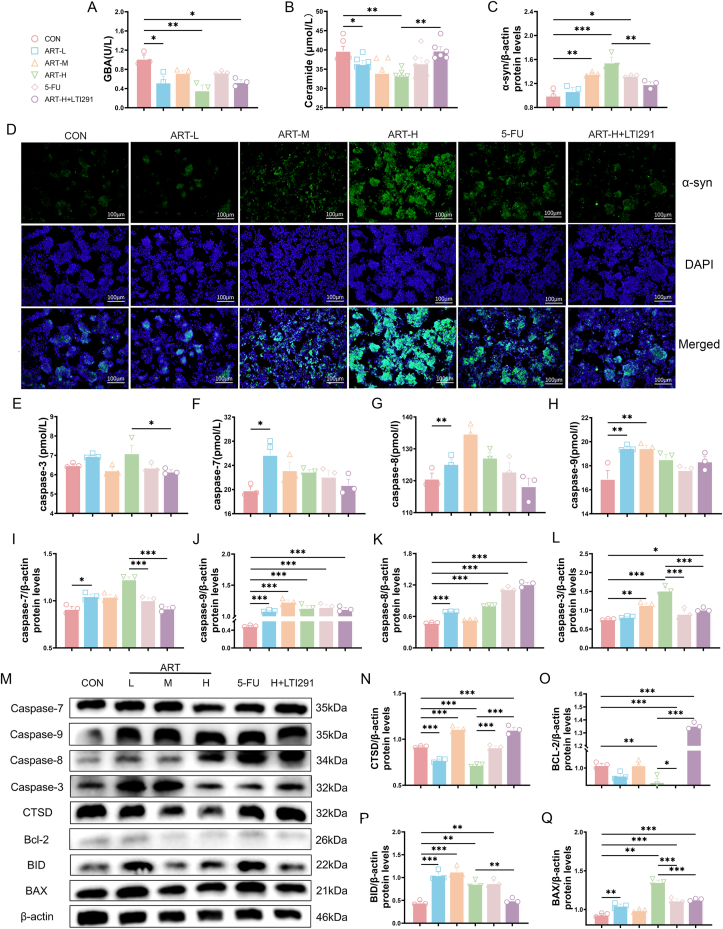
Regulatory effects of artesunate on GBA-Ceramide-CTSD-BID-BAX signaling in HepG2 cells (*n* = 3). **(A)** GBA (ELISA). **(B)** Ceramide (ELISA). **(C, D)** α-syn (immunofluorescence). Scale bars, 100 μm. **(E**–**H)** Caspase-3, Caspase-7, Caspase-8, Caspase-9 (ELISA). **(I**–**Q)** Western blotting analysis. (M) Western blots. (I) Caspase-7. (J) Caspase-9. (K) Caspase-8. (L) Caspase-3. (N) CTSD. (O) BCL-2. (P) BID. (Q) BAX. CON: HepG2 control group. ART-L, ART-M, ART-H: low-, middle-, and high-dose of artesunate treatment groups. 5-FU: positive drug 5-fluorouracil treatment group. ART-H + LTI-291: high dose of artesunate and GBA enzyme activator LTI-291 treatment group. Data were expressed as mean ± standard deviation. *∗P* < 0.05, ∗∗*P* < 0.01, and ∗∗∗*P* < 0.001.

**Figure 5:**
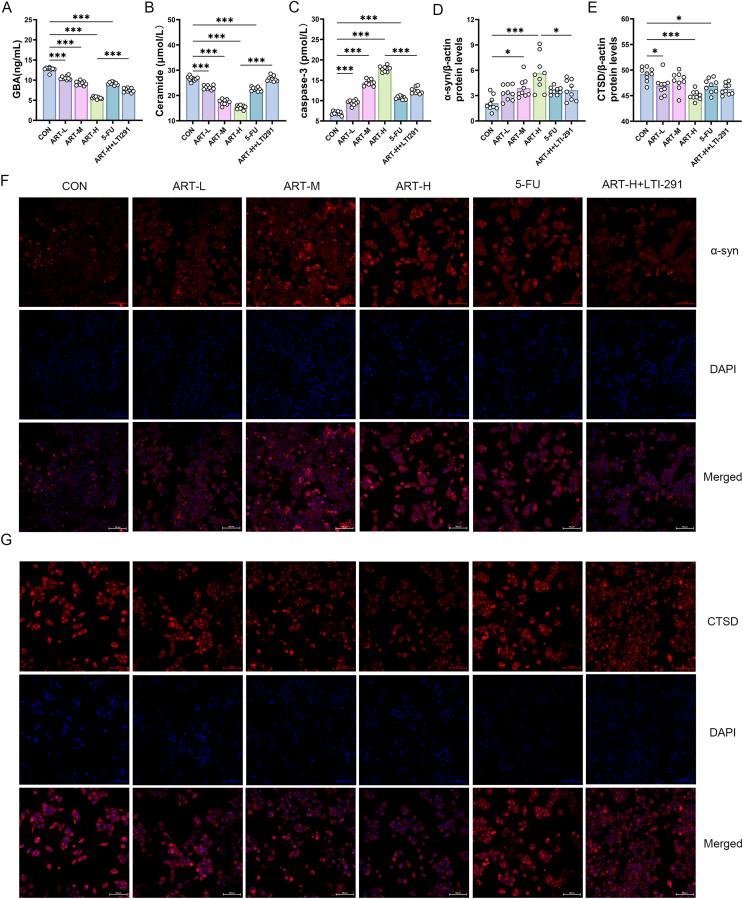
Regulatory effects of artesunate on GBA-Ceramide-CTSD-BID-BAX signaling in MHCC-97H cells (*n* = 3). **(A)** GBA (ELISA). **(B)** Ceramide (ELISA). **(C)** Caspase-3. **(D, F)** α-syn (immunofluorescence). Scale bars, 100 μm. **(E, G)** CTSD (IF). Scale bars, 100 μm. CON: MHCC-97H control group. ART-L, ART-M, ART-H: low-, middle-, and high-dose of artesunate treatment groups. 5-FU: positive drug 5-fluorouracil treatment group. ART-H + LTI-291: high dose of artesunate and GBA enzyme activator LTI-291 treatment group. Data were expressed as mean ± standard deviation. *∗P* < 0.05, ∗∗*P* < 0.01, and ∗∗∗*P* < 0.001.

*In vitro* research using HepG2 cells consistently recapitulated the significant modulatory effects of artesunate on the apoptosis-associated GBA-ceramide-CTSD-BID-BAX signaling pathway. Briefly, artesunate inhibited the activities of GBA and ceramide, reduced the CTSD protein expression levels, aggregated the expression of α-syn, which initiates the apoptosis bioprocess by elevating the expression of the apoptosis promoters caspase-3, caspase-7, caspase-8, caspase-9, BID, and BAX, and decreased that of the anti-apoptotic protein apoptosis regulator BCL-2, whose effects were similar to those of the positive drug 5-fluorouracil and impaired by LTI-291 (Fig. 4). Consistent with the findings in HepG2 cells, artesunate treatment in MHCC-97H cells suppressed GBA enzyme activity, leading to a subsequent reduction in CTSD expression and a reduction of ceramide. These changes were associated with increased α-synuclein accumulation and elevated caspase-3 enzyme activity (Fig. 5).

### Y313, E340, and N396 function as pivotal binding sites in the binding of artesunate to GBA

**Figure 6:**
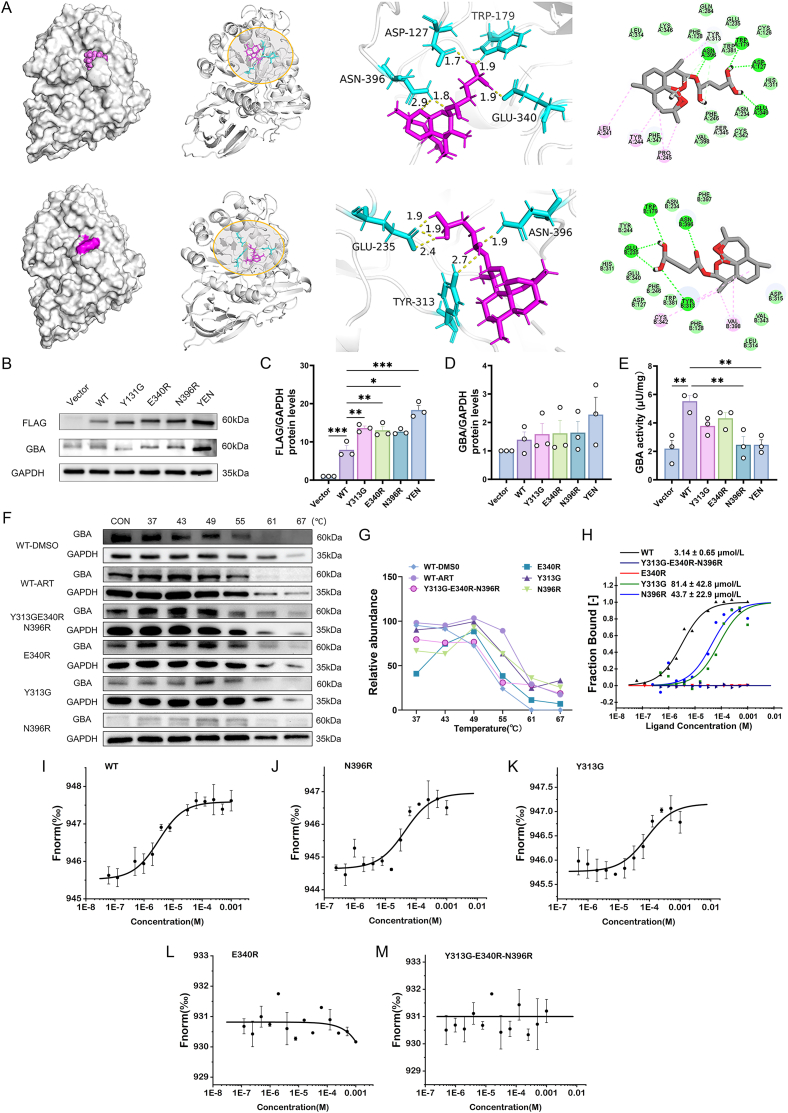
Identification of important binding sites for artesunate with GBA protein (*n* = 3). **(A)** Two- and three-dimensional images of artesunate with the binding pocket that formed with the amino acids of GBA and their interacting conventional hydrogen bonds using AutoDock Vina, Ledock, and Schrödinger molecular docking. **(B)** The expression levels of the FLAG tag and GBA protein were detected by Western blotting. **(C, D)** Quantitative results for FLAG and GBA protein expression levels are shown. **(E)** GBA enzymatic activity was measured. **(F)** Cellular thermal shift assay (CETSA)-Western blotting experiment detected the thermal stability of wild-type GBA and mutant-type GBA (Y313G, E340R, N396R, Y313G/E340R/N396R) plasmid mutant proteins after treatment with 29.27 μM artesunate. **(G)** The CETSA curve and the thermal stability to reach the Tm 50 value were performed using GraphPad Prism software. **(H**–**M)** Detection for binding affinities of artesunate with wild-type GBA and mutant-type GBA (Y313G, E340R, N396R, Y313G/E340R/N396R) using microscale thermophoresis (MST) assay. **(H)** Dose–response curves for calculating binding affinities between artesunate and wild-type GBA and mutant-type GBA (Y313G, E340R, N396R, Y313G/E340R/N396R) by MST. **(I)** Artesunate with wide-type GBA. **(J)** Artesunate with mutant-type GBA (N396R). **(K)** Artesunate with mutant-type GBA (Y313G). **(L)** Artesunate with mutant-type GBA (E340R). **(M)** Artesunate with mutant-type GBA (Y313G/E340R/N396R). WT-DMSO: wide-type GBA and DMSO group. WT-ART: wide-type GBA and 29.27 μM artesunate treatment group. Y313G-E340R–N396R-ART, E340R-ART, Y313G-ART, and N396R-ART: mutant-type GBA (Y313G/E340R/N396R, E340R, Y313G, N396R).

Computational docking models were constructed, and the docking results demonstrated that the binding affinity of artesunate for GBA was −8.7 kcal/mol. Two-dimensional images showed that artesunate bound to a binding pocket formed by the amino acids of GBA, and the three-dimensional images showed that the artesunate complex formed five conventional hydrogen bonds with no. 340 glutamic acid (GLU340), no. 396 asparagine (ASN396), no. 179 tryptophan (TRP179), no. 127 aspartic acid (ASP127), and no. 313 tyrosine (TYR313) of the GBA protein with an occurrence frequency of 13, 13, 15, 13, and 7, respectively, to stabilize the binding conformations (Fig. 6A). TYR313 is essential for catalysis and is an active site for GBA.^28^ Detailed information on the docking results of artesunate with GBA using AutoDock Vina, LeDock, and Schrödinger is listed in Table S3. Alanine scanning was used to analyze the GBA peptides by systematically substituting the residues with alanine; consequently, the alanine-tolerant residues can be subjected to structural modifications without impairing their bioactivity. The amino acids GLU340, ASN396, TRP179, and ASP127 were substituted with alanine, starting at the C-terminus and ending at the N-terminus. Decreased binding affinities were observed between artesunate and GBA for GLU340, ASN396, and TRP179, whereas only a slight difference in binding affinity was observed for ASP127. Then, both one-point saturation mutation (OPM) and multi-point saturation mutations (MPM) were performed for these residues, revealing that the mutational energies were the highest when GLU340 (OPM: 0.96 kcal/mol; MPM: 1.88 kcal/mol) and ASN396 (OPM: 0.67 kcal/mol; MPM: 5.22 kcal/mol) were mutated to arginine, and TYR313 (OPM: 0.94 kcal/mol; MPM: 3.59 kcal/mol) was mutated to glycine based on the compound–protein complexes with the receptors 6Q6N, 6Q6L, 6MOZ, 2V3D, 2XWE, and 6Q6K. Detailed information on alanine scanning and saturation mutations in the GLU340, ASN396, TYR313, and TRP179 of GBA, which was used to assess their binding affinities with artesunate, is provided in Table S4. The results of the molecular docking and mutational analyses indicate that mutations in Y313G, E340R, and N396R, the most important interacting amino acids of GBA, can positively affect the ability of artesunate to halt HCC development.

Then, the expression of the transfected wild-type and mutant GBA proteins was confirmed by Western blotting analysis using an anti-FLAG antibody. The results demonstrated comparable and robust expression levels across all constructs (Fig. 6B–D). We next measured the enzymatic activity of GBA in these transfected cells. As shown in Figure 6E, the wild-type GBA displayed the highest activity. In contrast, all mutant forms of GBA, particularly the Y313G/E340R/N396R triple mutant, exhibited a significant reduction in enzymatic activity. These findings collectively indicate that the Y313, E340, and N396 residues are critical not only for maintaining GBA's catalytic function but also for its structural integrity in binding artesunate.

To confirm the virtual simulation results, cellular thermal shift assay with HepG2 cells and microscale thermophoresis was performed after the reaction with wild-type and mutant-type GBA (Y313G, E340R, N396R, and Y313G/E340R/N396R). The cellular thermal shift assay data revealed that, compared with the wild-type DMSO group, the rates of degradation of GBA proteins increased with the temperature in the mutant-type GBA (E340R, Y313G/E340R/N396R) groups. In contrast, the degradation rates in the wild-type artesunate and mutant-type GBA (Y313G, N396R) groups were relatively low, with a hot melting curve shift to the right side. These findings indicate that the mutations in Y313G, E340R, and N396R, especially E340R, largely impaired the thermal stability of GBA with artesunate and were responsible for their interplay, serving as key binding sites (Fig. 6F and G), which was further verified using the microscale thermophoresis assay. As expected, artesunate readily bound to wild-type GBA, with an estimated K_d_ of 3.14 μmol/L. To verify the important role of Y313, E340, and N396 in this complex interaction, cell lysates of mutant-type GBA (Y313G, E340R, N396R, and Y313G/E340R/N396R) were obtained by constructing the corresponding eukaryotic expression plasmids.^29^^,^^30^ As shown in Figure 6H–M, the binding affinities of artesunate with the mutant-type GBA (Y313G and N396R) tended to be significantly lower, with estimated K_d_ values of 81.4 and 43.7 μmol/L, respectively, and that of artesunate with the mutant-type GBA (E340R and Y313G/E340R/N396R) did not generate binding curves. These results confirm that Y313, E340, and N396 are important binding sites for artesunate to bind to the GBA protein in HCC cells.

### Mutations in the key binding sites of the GBA protein abolish the modulatory effects of artesunate on the GBA-Ceramide-CTSD-BID-BAX apoptosis-related signaling

**Figure 7:**
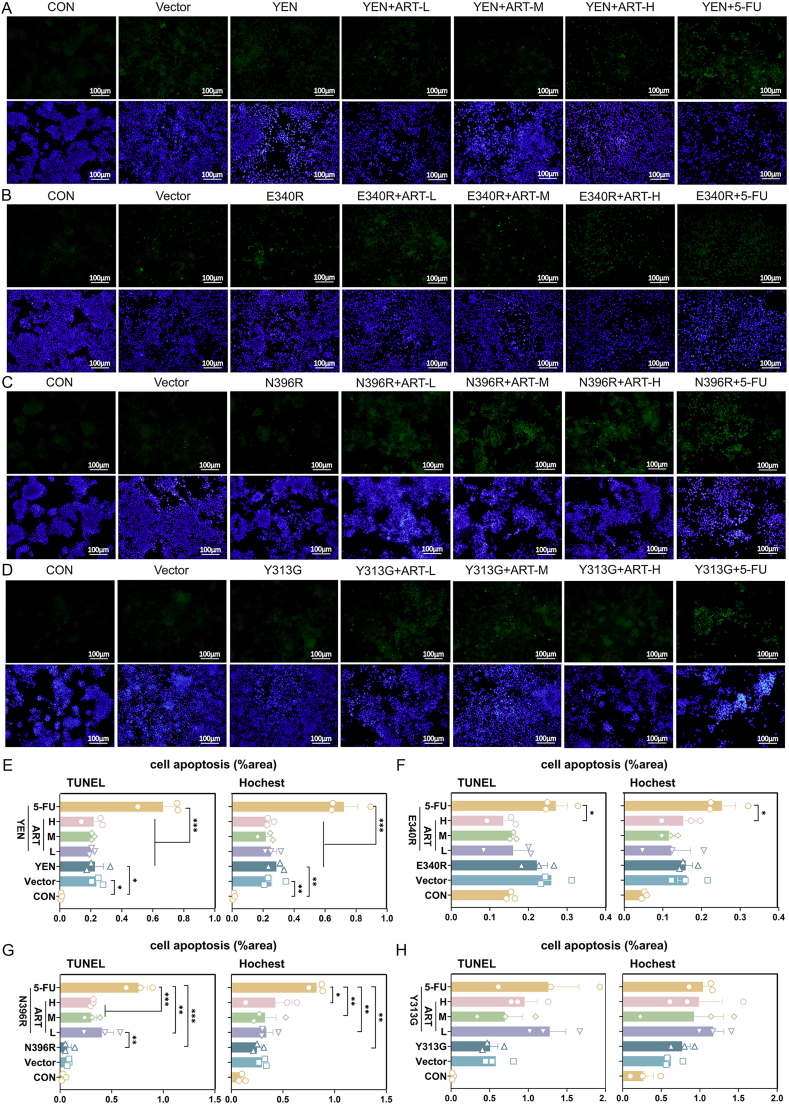
Effects of artesunate on the apoptosis of HepG2 cells after mutating GBA (Y313G, E340R, N396R, Y313G/E340R/N396R) detected by TUNEL and Hoechst staining (*n* = 3). **(A, E)** Apoptosis analysis for mutant-type GBA (Y313G/E340R/N396R) after 29.27 μM artesunate treatment. Scale bars, 100 μm. **(B, F)** Apoptosis analysis for mutant-type GBA (E340R) after 29.27 μM artesunate treatment. Scale bars, 100 μm. **(C, G)** Apoptosis analysis for mutant-type GBA (N396R) after 29.27 μM artesunate treatment. Scale bars, 100 μm. **(D, H)** Apoptosis analysis for mutant-type GBA (Y313G) after 29.27 μM artesunate treatment. Scale bars, 100 μm. The percentage of apoptotic cells derived from at least five random fields of view (*n* = 3) was analyzed by two independent observers who were blinded to the experimental groups. YEN, E340R, N396R, Y313G: mutant-type GBA (Y313G/E340R/N396R, E340R, N396R, Y313G). ART-L, ART-M, ART-H: low-, middle-, high-dose of artesunate treatment. 5-FU: positive drug 5-fluorouracil treatment. Data were expressed as mean ± standard deviation. *∗P* < 0.05, ∗∗*P* < 0.01, and ∗∗∗*P* < 0.001.

**Figure 8:**
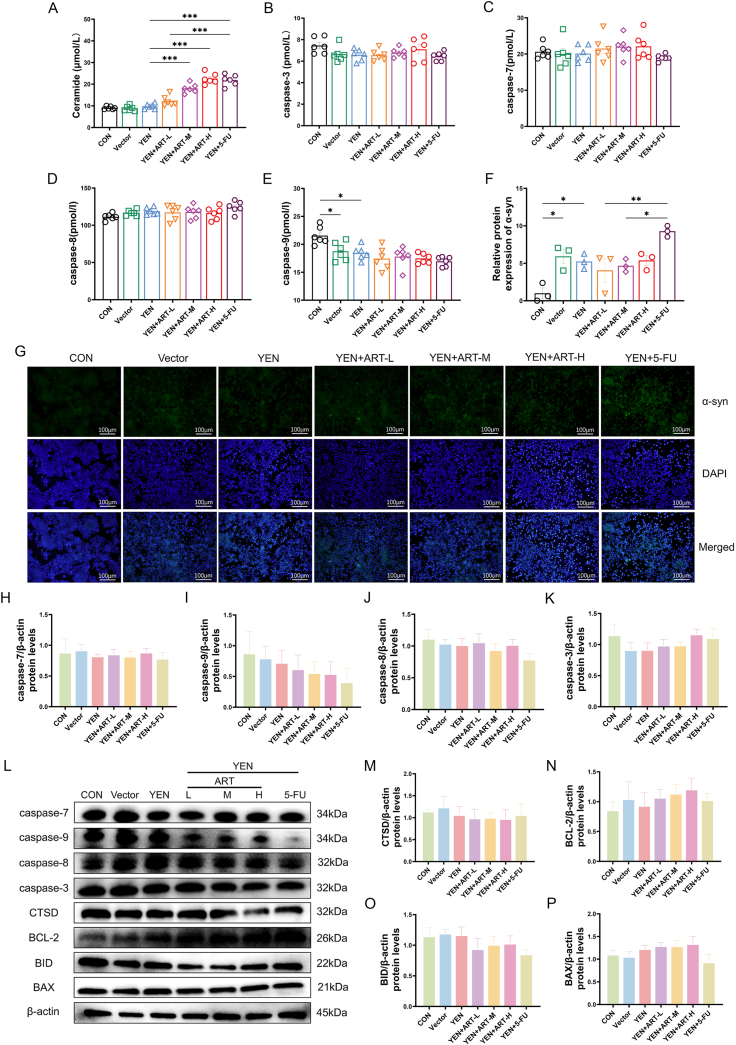
Effects of artesunate on GBA-Ceramide-CTSD-BID-BAX signaling in HepG2 cells after mutating GBA (Y313G, E340R, N396R, Y313G/E340R/N396R) (*n* = 3). **(A)** Ceramide (ELISA). **(B)** Caspase-3 (ELISA). **(C)** Caspase-7 (ELISA). **(D)** Caspase-8 (ELISA). **(E)** Caspase-9 (ELISA). **(F, G)** α-syn (immunofluorescence). Scale bars, 100 μm. **(H–P)** Western blotting analysis. **(L)** Western blots. (H) Caspase-7. (I) Caspase-9. (J) Caspase-8. (K) Caspase-3. (M) CTSD. (N) BCL-2. (O) BID. (P) BAX. YEN: mutant-type GBA (Y313G/E340R/N396R). ART-L, ART-M, ART-H: low-, middle-, high-dose of artesunate treatment. 5-FU: positive drug 5-fluorouracil treatment. Data were expressed as mean ± standard deviation. *∗P* < 0.05, ∗∗*P* < 0.01, and ∗∗∗*P* < 0.001.

To further verify the essential roles of the three key binding sites (Y313, E340, and N396) of GBA in the regulatory effects of artesunate against HCC, we determined the efficacy of artesunate treatment after mutating the binding sites of GBA by designing and synthesizing the corresponding plasmids. Consequently, no statistical significance exists in the basal apoptosis rate of all GBA1 mutants compared with wild-type GBA1 without drug intervention (Fig. S1). Mutating GBA using Y313G, E340R, N396R, and Y313G/E340R/N396R pCDNA3.1 plasmids deadly reversed the cell apoptosis sensitivity of artesunate, and no differences were found between these groups and the vector group; however, the positive drug 5-fluorouracil led to significant differences (Fig. 7). Moreover, artesunate failed to induce the activation of the apoptosis-associated GBA-ceramide-CTSD-BID-BAX signaling pathway without affecting the enzymatic activities and expression levels of the apoptosis promoters caspase-3, caspase-7, caspase-8, and caspase-9, or the protein expression levels of CTSD and apoptotic markers (BID, BAX, and BCL-2) (Fig. 8). These data indicate that mutations in the key binding sites of GBA, Y313, E340, and N396, may induce the loss or suppression of artesunate activity against HCC and play essential roles in aggravating artesunate-mediated apoptosis signaling.

## Discussion

HCC remains a malignancy with limited therapeutic options, driving the need for novel, effective interventions. Artesunate, a semi-synthetic derivative of artemisinin, has emerged as a promising anti-cancer agent with demonstrated efficacy against various solid tumors, including HCC.^31^ Its anti-HCC potential has been attributed to multiple mechanisms, such as disruption of iron homeostasis^32^ and augmentation of γδ T cell-mediated anti-tumor immunity.^33^ Previous work from our group identified GBA as a direct molecular target of artesunate and revealed its role in inhibiting lysosomal autophagy in HCC.^9^ However, the precise structural basis of the artesunate–GBA interaction and the specific downstream apoptotic signaling pathway remained incompletely characterized. This study builds upon that foundation to provide a more comprehensive mechanistic understanding, significantly advancing beyond prior reports.

Our integrated approach, combining molecular docking with cellular thermal shift assay and microscale thermophoresis, precisely delineates the artesunate-binding interface on GBA. We demonstrate that artesunate binds with high affinity, forming a stabilizing network of conventional hydrogen bonds with key residues, including TYR313, GLU340, and ASN396. Functional mutagenesis revealed the distinct contributions of these residues: while the N396R mutation most severely compromised GBA's intrinsic enzymatic activity, the E340R mutation most dramatically impaired artesunate binding while largely preserving basal catalytic function. This critical dissociation identifies GLU340 as a specific anchor point for artesunate and suggests an allosteric mechanism of inhibition, as the drug-binding site does not fully overlap with the enzyme's catalytic core. The functional consequence was confirmed in apoptosis assays, where the pro-apoptotic effect of artesunate correlated directly with binding affinity, being most potent in wild-type GBA cells and virtually abolished in E340R mutants. This constitutes a key novel finding, moving beyond target identification to define the precise chemical interactions required for artesunate's anti-HCC activity.

Beyond target engagement, we mechanistically dissected the downstream signaling events. Guided by network analysis that enriched GBA-associated genes in lysosomal and sphingolipid metabolism, we proposed and experimentally validated a coherent GBA-ceramide-CTSD-α-syn-BID-BAX apoptotic axis. Our data indicate that artesunate-induced GBA inhibition reduces ceramide levels, which in turn impairs the maturation and function of CTSD, leading to the accumulation of α-syn. The accumulated α-syn subsequently triggers the mitochondrial apoptotic pathway by up-regulating BAX and facilitating BID cleavage, culminating in caspase activation. Crucially, rescue experiments confirmed that both ceramide supplementation and α-syn inhibition attenuated artesunate-induced apoptosis. This work provides the first evidence linking artesunate's engagement of GBA to the induction of mitochondrial apoptosis via this defined metabolic and proteostatic cascade in HCC.

Our findings possess significant translational relevance. First, the elucidated mechanism provides a plausible explanation for the observed synergy between artesunate and sorafenib,^34^ suggesting combination therapy as a viable clinical strategy. Second, given that GBA is highly expressed in HCC and associated with patient prognosis,^35^ it constitutes a compelling therapeutic target. Most importantly, the identification of critical binding residues, particularly GLU340, provides a precise molecular blueprint for rational drug design. Future efforts can leverage this structural insight to develop next-generation artesunate derivatives or novel small molecules with optimized binding affinity and specificity for GBA, potentially leading to more potent and targeted HCC therapies.

We acknowledge several limitations that also outline future research directions. Firstly, the pathway was rigorously validated primarily in two HCC cell lines. Its generalizability across the molecular heterogeneity of HCC should be confirmed in broader panels of cell models or, ideally, in patient-derived organoids. Secondly, while no acute toxicity was observed, a systematic preclinical assessment of chronic toxicity is warranted to fully establish its safety profile for potential long-term HCC management. Future work should prioritize validating this mechanistic pathway in patient-derived xenograft models, which would more accurately recapitulate tumor heterogeneity and microenvironment, thereby strengthening the clinical relevance of our findings and facilitating their translation.

## Conclusion

**Figure 9:**
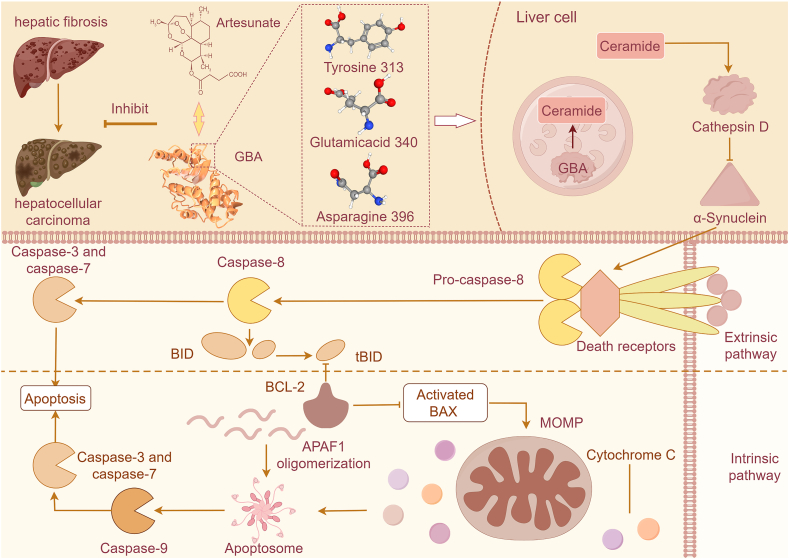
Mechanism diagram depicting that GBA-Ceramide-CTSD-BID-BAX signaling may be the key putative target pathway of artesunate in halting the progression of HCC, and Y313, E340, and N396 may serve as important binding sites of GBA protein with artesunate (By Figdraw).

Our study provides a comprehensive mechanistic elucidation of artesunate's anti-HCC activity. We have not only confirmed GBA as a direct target but have precisely mapped its binding interface, identifying TYR313, GLU340, and ASN396 as critical residues for artesunate engagement. Functional dissection revealed a functional divergence among these residues, with GLU340 serving as the primary anchor for drug binding and ASN396 being indispensable for catalytic activity, suggesting an allosteric inhibition mechanism. Furthermore, we delineated a novel GBA-ceramide-CTSD-α-syn-BID-BAX signaling axis, thereby connecting artesunate's target engagement to the execution of mitochondrial apoptosis (Fig. 9). These findings significantly advance our understanding beyond previous studies and provide a robust structural and mechanistic foundation for the rational development of GBA-targeted therapies for HCC.

## CRediT authorship contribution statement

**Xia Mao:** Writing – original draft, Methodology, Investigation. **Xiangying Yan:** Methodology, Investigation, Data curation. **Yawen Chen:** Visualization. **Bingbing Cai:** Visualization. **Wenjia Chen:** Visualization. **Ya Lin:** Visualization. **Na Lin:** Supervision, Resources. **Yanqiong Zhang:** Writing – review & editing, Supervision, Conceptualization.

## Ethics declaration

Animal experiments were conducted according to the Guidelines for the Care and Use of Laboratory Animals of the Animal Ethics and Welfare Committee, Guangzhou Forevergen Medical Laboratory Animal Center, Guangdong, China (license no. IACUCAEWC-F2005003).

## Funding

This study is funded by the National Natural Science Foundation of China (No. 82104467, 82574854), National Science and Technology Major Project for the Prevention and Control of Emerging and Major Infectious Diseases (No. 2025ZD01906000, 2025ZD01906004), Scientific and Technological Innovation Project of China Academy of Chinese Medical Sciences (No. CI2023E001TS02, CI2023E001TS06, CI2023E002-Y-51), and Fundamental Research Funds for the Central Public Welfare Research Institutes (China) (No. ZZ15-YQ-029, ZXKT22042, ZXKT25035). No study sponsors are involved in the research process of this project.

## Conflict of interests

The authors declared no competing interests.
