# Supplementary material for: Artesunate directly targets glucosylceramidase to suppress hepatocellular carcinoma proliferation and trigger apoptosis
Source: Genes Dis. 2026 Jan 20;13(5):102045. doi: 10.1016/j.gendis.2026.102045 (PMC13265689; doi:10.1016/j.gendis.2026.102045)
Supplement: Multimedia component 1 [file mmc1.docx]

**Supplementary Files**

**Supplementary Materials** **Section 1.** Surface plasmon resonance detection

**Supplementary Materials-Table S1.** Detailed information of antibodies to caspase-3, -7, -8, -9, CTSD, BID, BAX, BCL-2 and GBA proteins

**Supplementary Materials-Table S2.** Detailed information of gene sequence and primer sequence for wide-type GBA1 and mutant-type GBA1 (TYR313GLY, GLU340ARG, ASN396ARG, TYR313GLY/GLU340ARG/ASN396ARG) used in microscale thermophoresis experiment

**Supplementary Materials-Table S3.** Detailed information of docking results of artesunate with GBA using AutoDock Vina, Ledock and Schrödinger

**Supplementary Materials-Table S4.** Detailed information of alanine scanning and saturation mutations for GLU340, ASN396, TYR313 and TRP179 in GBA for assessing their binding affinities with artesunate

**Supplementary Materials-Figure S1**. Quantitative analysis of apoptosis in HepG2 cells expressing GBA point mutants (Y313G, E340R, N396R, Y313G/E340R/N396R) in response to artesunate treatment (n=3)

**Section 1. Surface plasmon resonance detection**

SPR detection was performed to confirm the binding affinities of GBA protein with artesunate and GBA inhibitor Conduritol B Epoxide (CBE)

A. Amine coupling GBA on CM5 chip

Using PBS-EP buffer (1 mM HEPES, 150 mM NaCl, 3 mM EDTA, 0.05% surfactant P20, pH 7.4) as the experimental running buffer, the 2-channel of the CM5 chip was selected by mixing the same volume of freshly 50 mM N-hydroxysuccinimide (NHS) and 200 mM 1-ethyl-3-( 3-dimethylaminopropyl) carbodiimide hydrochloride (EDC). This mixture was injected into channel 2 at a flow rate of 10 μL/min for 600 s. GBA was diluted 10-fold with 10 mM acetate 4.0 and coupled to channel 2 of the CM5 chip at a level of approximately 14,000 Ru. The remaining activation sites were blocked by injecting 1 M ethanolamine for 420 s at a flow rate of 10 μL/min. Equilibrating the chip with well-coupled proteins at a flow rate of 10 μL/min.

B. Determining the affinity of GBA with artesunate and CBE

The binding affinity of GBA to small molecules was determined in each injection cycle using PBS-EP as experimental buffer. One channel was used as a blank reference channel and Two channels captured proteins. Diluted small molecules were injected sequentially into channels 1 and 2 at a concentration of 100-0.390 uM at a flow rate of 30 μL/min, with a binding time of 90 s and a dissociation time of 420 s. The binding time of GBA and the dissociation time of GBA were measured with Glycine 4.5 at a flow rate of 30 μL/min. Glycine 4.5 was injected at a flow rate of 30 µL/min for 60 s to complete the microarray regeneration experiments at a temperature of 25℃. The microarray regeneration experiments were performed at a temperature of 25 ℃. The Biacore T200 evaluation software 2.0 (GE Healthcare) was used for calculating the related K_a_, K_d_, and K_D_ values. Especially, the equilibrium dissociation constant K_D_ was obtained from the ratio of the dissociation rate constant K_d_ divided by the association rate constant Ka (K_D_=K_d_/K_a_).

**Supplementary Materials-Table S1.**

**Detailed information of antibodies to caspase-3, -7, -8, -9, CTSD, BID, BAX and GBA proteins**

| **Antibodies** | **Molecular weight** | **Cat No.** | **Sources** | **Concentration** | **Companies** |
| --- | --- | --- | --- | --- | --- |
| Caspase-3 | 32 kDa | 19677-1-AP | Rabbit | 1:2000 | Proteintech Group Inc, Wuhan, China |
| Caspase-7 | 35 kDa | 27155-1-AP | Rabbit | 1:1000 | Proteintech Group Inc, Wuhan, China |
| Caspase-8 | 34 kDa | 13423-1-AP | Rabbit | 1:2000 | Proteintech Group Inc, Wuhan, China |
| Caspase-9 | 35 kDa | 10380-1-AP | Rabbit | 1:2000 | Proteintech Group Inc, Wuhan, China |
| Cathepsin D | 32 kDa | 21327-1-AP | Rabbit | 1:2000 | Proteintech Group Inc, Wuhan, China |
| BH3-interacting domain death agonist | 22 kDa | 10988-1-AP | Rabbit | 1:2000 | Proteintech Group Inc, Wuhan, China |
| BCL2-associated X | 21 kDa | 60267-1-lg | Mouse | 1:1000 | Proteintech Group Inc, Wuhan, China |
| B-cell lymphoma 2 | 26 kDa | 26593-1-AP | Rabbit | 1:1000 | Proteintech Group Inc, Wuhan, China |
| Glucosylceramidase | 60 kDa | AB128879 | Rabbit | 1:1000 | Abcam  Cambridge, UK |
| Glyceraldehyde 3-phosphate dehydrogenase | 36 kDa | 60004-1-lg | Mouse | 1:10000 | Proteintech Group Inc, Wuhan, China |
| β actin | 46 kDa | 4970S | Rabbit | 1:10000 | Cell signaling technology  Boston, USA |
| Horseradish peroxidase-conjugated anti-rabbit IgG | --- | BA1054 | --- | 1:3000 | Boster, Wuhan, China |
| Horseradish peroxidase-conjugated anti-mouse IgG | --- | BA1050 | --- | 1:3000 | Boster, Wuhan, China |

**Supplementary Materials-Table S2.**

**Detailed information of gene sequence and primer sequence for wide-type GBA1 and mutant-type GBA1 (TYR313GLY, GLU340ARG, ASN396ARG, TYR313GLY/GLU340ARG/ASN396ARG) used in microscale thermophoresis experiment**

| **Primer/Plasmids** | **Sequence information** |
| --- | --- |
| Primer | PEGFP-C-5 CATGGTCCTGCTGGAGTTCGTG; PFastBac1-R TTCAGGTTCAGGGGGAGGTG  G42120-1-seq1 AGTTGGCCCAGCGTCCCGTT |
| EGFP-GBA1(wide-type)-6^*^His | MVSKGEELFTGVVPILVELDGDVNGHKFSVSGEGEGDATYGKLTLKFICTTGKLPVPWPTLVTTLTYGVQCFSRYPDHMKQHDFFKSAMPEGYVQERTIFFKDDGNYKTRAEVKFEGDTLVNRIELKGIDFKEDGNILGHKLEYNYNSHNVYIMADKQKNGIKVNFKIRHNIEDGSVQLADHYQQNTPIGDGPVLLPDNHYLSTQSALSKDPNEKRDHMVLLEFVTAAGITLGMDELYK-ARPCIPKSFGYSSVVCVCNATYCDSFDPPTFPALGTFSRYESTRSGRRMELSMGPIQANHTGTGLLLTLQPEQKFQKVKGFGGAMTDAAALNILALSPPAQNLLLKSYFSEEGIGYNIIRVPMASCDFSIRTYTYADTPDDFQLHNFSLPEEDTKLKIPLIHRALQLAQRPVSLLASPWTSPTWLKTNGAVNGKGSLKGQPGDIYHQTWARYFVKFLDAYAEHKLQFWAVTAENEPSAGLLSGYPFQCLGFTPEHQRDFIARDLGPTLANSTHHNVRLLMLDDQRLLLPHWAKVVLTDPEAAKYVHGIAVHWYLDFLAPAKATLGETHRLFPNTMLFASEACVGSKFWEQSVRLGSWDRGMQYSHSIITNLLYHVVGWTDWNLALNPEGGPNWVRNFVDSPIIVDITKDTFYKQPMFYHLGHFSKFIPEGSQRVGLVASQKNDLDAVALMHPDGSAVVVVLNRSSKDVPLTIKDPAVGFLETISPGYSIHTYLWRRQ-HHHHHH |
| EGFP-GBA1(mutant-type, TYR313GLY)-6^*^His | MVSKGEELFTGVVPILVELDGDVNGHKFSVSGEGEGDATYGKLTLKFICTTGKLPVPWPTLVTTLTYGVQCFSRYPDHMKQHDFFKSAMPEGYVQERTIFFKDDGNYKTRAEVKFEGDTLVNRIELKGIDFKEDGNILGHKLEYNYNSHNVYIMADKQKNGIKVNFKIRHNIEDGSVQLADHYQQNTPIGDGPVLLPDNHYLSTQSALSKDPNEKRDHMVLLEFVTAAGITLGMDELYK-ARPCIPKSFGYSSVVCVCNATYCDSFDPPTFPALGTFSRYESTRSGRRMELSMGPIQANHTGTGLLLTLQPEQKFQKVKGFGGAMTDAAALNILALSPPAQNLLLKSYFSEEGIGYNIIRVPMASCDFSIRTYTYADTPDDFQLHNFSLPEEDTKLKIPLIHRALQLAQRPVSLLASPWTSPTWLKTNGAVNGKGSLKGQPGDIYHQTWARYFVKFLDAYAEHKLQFWAVTAENEPSAGLLSGYPFQCLGFTPEHQRDFIARDLGPTLANSTHHNVRLLMLDDQRLLLPHWAKVVLTDPEAAKYVHGIAVHWGLDFLAPAKATLGETHRLFPNTMLFASEACVGSKFWEQSVRLGSWDRGMQYSHSIITNLLYHVVGWTDWNLALNPEGGPNWVRNFVDSPIIVDITKDTFYKQPMFYHLGHFSKFIPEGSQRVGLVASQKNDLDAVALMHPDGSAVVVVLNRSSKDVPLTIKDPAVGFLETISPGYSIHTYLWRRQ-HHHHHH |
| EGFP-GBA1(mutant-type, GLU340ARG)-6^*^His | MVSKGEELFTGVVPILVELDGDVNGHKFSVSGEGEGDATYGKLTLKFICTTGKLPVPWPTLVTTLTYGVQCFSRYPDHMKQHDFFKSAMPEGYVQERTIFFKDDGNYKTRAEVKFEGDTLVNRIELKGIDFKEDGNILGHKLEYNYNSHNVYIMADKQKNGIKVNFKIRHNIEDGSVQLADHYQQNTPIGDGPVLLPDNHYLSTQSALSKDPNEKRDHMVLLEFVTAAGITLGMDELYK-ARPCIPKSFGYSSVVCVCNATYCDSFDPPTFPALGTFSRYESTRSGRRMELSMGPIQANHTGTGLLLTLQPEQKFQKVKGFGGAMTDAAALNILALSPPAQNLLLKSYFSEEGIGYNIIRVPMASCDFSIRTYTYADTPDDFQLHNFSLPEEDTKLKIPLIHRALQLAQRPVSLLASPWTSPTWLKTNGAVNGKGSLKGQPGDIYHQTWARYFVKFLDAYAEHKLQFWAVTAENEPSAGLLSGYPFQCLGFTPEHQRDFIARDLGPTLANSTHHNVRLLMLDDQRLLLPHWAKVVLTDPEAAKYVHGIAVHWYLDFLAPAKATLGETHRLFPNTMLFASRACVGSKFWEQSVRLGSWDRGMQYSHSIITNLLYHVVGWTDWNLALNPEGGPNWVRNFVDSPIIVDITKDTFYKQPMFYHLGHFSKFIPEGSQRVGLVASQKNDLDAVALMHPDGSAVVVVLNRSSKDVPLTIKDPAVGFLETISPGYSIHTYLWRRQ-HHHHHH |
| EGFP-GBA1(mutant-type, ASN396ARG)-6^*^His | MVSKGEELFTGVVPILVELDGDVNGHKFSVSGEGEGDATYGKLTLKFICTTGKLPVPWPTLVTTLTYGVQCFSRYPDHMKQHDFFKSAMPEGYVQERTIFFKDDGNYKTRAEVKFEGDTLVNRIELKGIDFKEDGNILGHKLEYNYNSHNVYIMADKQKNGIKVNFKIRHNIEDGSVQLADHYQQNTPIGDGPVLLPDNHYLSTQSALSKDPNEKRDHMVLLEFVTAAGITLGMDELYK-ARPCIPKSFGYSSVVCVCNATYCDSFDPPTFPALGTFSRYESTRSGRRMELSMGPIQANHTGTGLLLTLQPEQKFQKVKGFGGAMTDAAALNILALSPPAQNLLLKSYFSEEGIGYNIIRVPMASCDFSIRTYTYADTPDDFQLHNFSLPEEDTKLKIPLIHRALQLAQRPVSLLASPWTSPTWLKTNGAVNGKGSLKGQPGDIYHQTWARYFVKFLDAYAEHKLQFWAVTAENEPSAGLLSGYPFQCLGFTPEHQRDFIARDLGPTLANSTHHNVRLLMLDDQRLLLPHWAKVVLTDPEAAKYVHGIAVHWYLDFLAPAKATLGETHRLFPNTMLFASEACVGSKFWEQSVRLGSWDRGMQYSHSIITNLLYHVVGWTDWNLALNPEGGPNWVRRFVDSPIIVDITKDTFYKQPMFYHLGHFSKFIPEGSQRVGLVASQKNDLDAVALMHPDGSAVVVVLNRSSKDVPLTIKDPAVGFLETISPGYSIHTYLWRRQ-HHHHHH |
| EGFP-GBA1(mutant-type, TYR313GLY, GLU340ARG, ASN396ARG)-6^*^His | MVSKGEELFTGVVPILVELDGDVNGHKFSVSGEGEGDATYGKLTLKFICTTGKLPVPWPTLVTTLTYGVQCFSRYPDHMKQHDFFKSAMPEGYVQERTIFFKDDGNYKTRAEVKFEGDTLVNRIELKGIDFKEDGNILGHKLEYNYNSHNVYIMADKQKNGIKVNFKIRHNIEDGSVQLADHYQQNTPIGDGPVLLPDNHYLSTQSALSKDPNEKRDHMVLLEFVTAAGITLGMDELYK-ARPCIPKSFGYSSVVCVCNATYCDSFDPPTFPALGTFSRYESTRSGRRMELSMGPIQANHTGTGLLLTLQPEQKFQKVKGFGGAMTDAAALNILALSPPAQNLLLKSYFSEEGIGYNIIRVPMASCDFSIRTYTYADTPDDFQLHNFSLPEEDTKLKIPLIHRALQLAQRPVSLLASPWTSPTWLKTNGAVNGKGSLKGQPGDIYHQTWARYFVKFLDAYAEHKLQFWAVTAENEPSAGLLSGYPFQCLGFTPEHQRDFIARDLGPTLANSTHHNVRLLMLDDQRLLLPHWAKVVLTDPEAAKYVHGIAVHWGLDFLAPAKATLGETHRLFPNTMLFASRACVGSKFWEQSVRLGSWDRGMQYSHSIITNLLYHVVGWTDWNLALNPEGGPNWVRRFVDSPIIVDITKDTFYKQPMFYHLGHFSKFIPEGSQRVGLVASQKNDLDAVALMHPDGSAVVVVLNRSSKDVPLTIKDPAVGFLETISPGYSIHTYLWRRQ-HHHHHH |

**Supplementary Materials-Table S3.**

**Detailed information of docking results of artesunate with GBA using AutoDock Vina, Ledock and Schrödinger**

| **PDB** | **Software** | **Docking score**  **(kcal/mol)** | **Binding sites** |
| --- | --- | --- | --- |
| 1Y7V | Schrodinger | -5.959 | ASN-396 ASP-127 TRP-179 GLU-235 ASN-234 |
|  | vina | -6.9 | ASN-396 ASN-234 GLU-340 |
|  | LEDOCK | -4.46 | ASP-127 TRP-179 TYR-313 TYR-244 |
| 6Q6N | Schrodinger | -7.159 | ASN-396 TYR-313 GLU-235 |
|  | vina | -7.3 | TYR-313 CYS-342 GLN-284 |
|  | LEDOCK | -4.84 | TYR-313 CYS-342 GLN-284 TYR-244 TRP-312 GLU-340 |
| 6Q6L | Schrodinger | -5.924 | ASP-127 TRP-381 GLU-235 ASN-234 TRP-179 ASN-396 |
|  | vina | -8.2 | TRP-381 ASN-396 GLU-340 |
|  | LEDOCK | -5.82 | ASP-127 ASN-234 TRP-179 ASN-396 TYR-244 |
| 6MOZ | Schrodinger | -6.437 | ASP-127 TRP-179 GLU-340 GLU-235 TYP-244 |
|  | vina | -8.6 | GLU-340 GLN-284 |
|  | LEDOCK | -6.2 | ASP-127 TRP-179 GLU-235 TYR-244 GLN-284 SER-345 TYR-313 ASN-234 |
| 2V3D | Schrodinger | -5.391 | GLU-340 ASP-127 TRP-179 ASN-396 |
|  | vina | -8.7 | GLU-340 TRP-179 TYR-244 SER-345 ASN-234 GLU-234 GLN-284 |
|  | LEDOCK | -5.81 | ASP-127 TRP-179 TYR-244 ASN-234 GLN-284 GLU-340 TYR-313 |
| 2XWE | Schrodinger | -6.184 | ASP-127 TRP-179 ASN-396 GLU-340 |
|  | vina | -8 | ASP-127 ASN-396 GLN-284 TYR-244 |
|  | LEDOCK | -5.38 | ASP-127 TRP-179 ASN-396 GLU-340 TYR-244 TRP-381 |
| 2XWD | Schrodinger | -6.793 | ASN-396 GLU-340 ASP-127 TRP-179 |
|  | vina | -7.9 | ASN-396 TYR-244 GLN-284 |
|  | LEDOCK | -5.49 | GLU-340 TRP-179 TYR-244 TYR-313 ASN-234 |
| 6Q6K | Schrodinger | -6.327 | ASN-396 ASP-127 TRP-381 TRP-179 GLU-235 |
|  | vina | -8.2 | GLU-235 |
|  | LEDOCK | -5.74 | TRP-381 TRP-179 GLU-235 GLU-340 ASN-234 |

**Supplementary Materials-Table S4.**

**Detailed information of alanine scanning and saturation mutations for GLU340, ASN396, TYR313 and TRP179 in GBA for assessing their binding affinities with artesunate**

**Part 1. Results of analysis using the 6Q6N PDB structure**

| Mutation | Mutation Energy  (kcal/mol) | Effect of Mutation | vDW Term | Electrostatic Term |
| --- | --- | --- | --- | --- |
| Alanine scanning | | | | |
| A:PHE128>ALA | -0.16 | NEUTRAL | 0 | -0.36 |
| A:ASP127>ALA | -0.08 | NEUTRAL | 1.63 | -1.86 |
| A:ASN234>ALA | 0.03 | NEUTRAL | 0.56 | -0.51 |
| A:VAL398>ALA | 0.07 | NEUTRAL | 0.01 | -0.04 |
| A:CYS342>ALA | 0.24 | NEUTRAL | 0.46 | -0.02 |
| A:TRP381>ALA | 0.26 | NEUTRAL | 0.73 | -0.28 |
| A:TYR244>ALA | 0.39 | NEUTRAL | 0.75 | -0.11 |
| A:ASN396>ALA | 0.51 | DESTABILIZING | 1.98 | -0.6 |
| A:TRP179>ALA | 0.55 | DESTABILIZING | 1.67 | -0.61 |
| A:PHE246>ALA | 0.84 | DESTABILIZING | 1.2 | 0.07 |
| A:GLU340>ALA | 0.85 | DESTABILIZING | 1.73 | -0.18 |
| A:TYR313>ALA | 1.6 | DESTABILIZING | 3.46 | -0.05 |
| Saturation mutagenesis | | | | |
| A:GLU340>TYR | -1.36 | STABILIZING | -2.45 | -0.26 |
| A:TRP179>LYS | -1.22 | STABILIZING | -1.18 | -1.27 |
| A:TYR313>TYR | -1.08 | STABILIZING | -2.18 | 0.06 |
| A:TYR313>TRP | -1.04 | STABILIZING | -2.28 | 0.08 |
| A:GLU340>HIS | -1 | STABILIZING | -1.59 | -0.37 |
| A:PHE246>LYS | -0.87 | STABILIZING | -1.96 | 0.15 |
| A:TRP179>TRP | -0.71 | STABILIZING | -1.45 | 0.03 |
| A:PHE246>PHE | -0.69 | STABILIZING | -1.44 | 0.05 |
| A:GLU340>PHE | -0.67 | STABILIZING | -1.33 | 0.01 |
| A:TYR313>PHE | -0.63 | STABILIZING | -1.14 | 0.03 |
| A:TRP179>HIS | -0.62 | STABILIZING | -0.63 | -0.63 |
| A:TYR313>GLN | -0.6 | STABILIZING | -2.12 | 0.48 |
| A:GLU340>LEU | -0.58 | STABILIZING | -1 | -0.17 |
| A:TRP179>MET | -0.54 | STABILIZING | -0.73 | -0.36 |
| A:TYR313>HIS | -0.44 | NEUTRAL | -0.84 | -0.08 |
| A:TRP179>LEU | -0.39 | NEUTRAL | -0.36 | -0.44 |
| A:PHE246>TRP | -0.39 | NEUTRAL | -1.33 | 0.72 |
| A:PHE246>HIS | -0.37 | NEUTRAL | -0.95 | 0.08 |
| A:TYR313>LEU | -0.3 | NEUTRAL | -0.57 | -0.04 |
| A:TRP179>GLN | -0.29 | NEUTRAL | -0.96 | 0.36 |
| A:PHE246>MET | -0.29 | NEUTRAL | -0.71 | 0.04 |
| A:TRP179>ILE | -0.27 | NEUTRAL | 0 | -0.56 |
| A:GLU340>ILE | -0.22 | NEUTRAL | -0.33 | -0.15 |
| A:PHE246>TYR | -0.19 | NEUTRAL | -0.54 | 0.22 |
| A:TRP179>THR | -0.17 | NEUTRAL | 0.28 | -0.66 |
| A:TYR313>ILE | -0.15 | NEUTRAL | -0.19 | -0.02 |
| A:TRP179>CYS | -0.1 | NEUTRAL | 0.38 | -0.61 |
| A:TRP179>TYR | -0.07 | NEUTRAL | -0.48 | 0.33 |
| A:TRP179>VAL | -0.04 | NEUTRAL | 0.5 | -0.61 |
| A:GLU340>GLU | -0.01 | NEUTRAL | -0.01 | 0 |
| A:GLU340>MET | -0.01 | NEUTRAL | 0.23 | -0.24 |
| A:GLU340>THR | -0.01 | NEUTRAL | 0.09 | -0.17 |
| A:GLU340>VAL | 0 | NEUTRAL | 0.15 | -0.19 |
| A:GLU340>LYS | 0.01 | NEUTRAL | -0.73 | 0.76 |
| A:TYR313>GLU | 0.02 | NEUTRAL | -1.79 | 1.54 |
| A:GLU340>ASN | 0.03 | NEUTRAL | 0.08 | -0.07 |
| A:GLU340>GLN | 0.05 | NEUTRAL | -0.07 | 0.18 |
| A:PHE246>LEU | 0.09 | NEUTRAL | -0.16 | 0.14 |
| A:GLU340>TRP | 0.14 | NEUTRAL | 0.43 | -0.18 |
| A:TRP179>PHE | 0.15 | NEUTRAL | 0.5 | -0.19 |
| A:TRP179>PRO | 0.16 | NEUTRAL | 0.95 | -0.66 |
| A:GLU340>SER | 0.17 | NEUTRAL | 0.36 | -0.15 |
| A:TRP179>GLU | 0.18 | NEUTRAL | -0.75 | 1.09 |
| A:PHE246>GLN | 0.18 | NEUTRAL | -0.29 | 0.53 |
| A:PHE246>ILE | 0.18 | NEUTRAL | 0.06 | 0.07 |
| A:GLU340>PRO | 0.25 | NEUTRAL | 0.53 | -0.16 |
| A:TRP179>ASP | 0.36 | NEUTRAL | 0.58 | 0.13 |
| A:PHE246>ARG | 0.36 | NEUTRAL | 0.17 | 0.45 |
| A:PHE246>ASN | 0.41 | NEUTRAL | 0.47 | 0.1 |
| A:TRP179>ARG | 0.46 | NEUTRAL | 1.92 | -1.05 |
| A:PHE246>GLU | 0.5 | NEUTRAL | -0.39 | 1.26 |
| A:TRP179>ASN | 0.53 | DESTABILIZING | 1.46 | -0.41 |
| A:TRP179>ALA | 0.55 | DESTABILIZING | 1.67 | -0.61 |
| A:PHE246>VAL | 0.59 | DESTABILIZING | 0.9 | -0.06 |
| A:GLU340>ASP | 0.59 | DESTABILIZING | 1.14 | -0.03 |
| A:PHE246>PRO | 0.6 | DESTABILIZING | 0.88 | -0.05 |
| A:TRP179>SER | 0.61 | DESTABILIZING | 1.77 | -0.59 |
| A:PHE246>THR | 0.62 | DESTABILIZING | 0.95 | -0.08 |
| A:TRP179>GLY | 0.63 | DESTABILIZING | 1.83 | -0.62 |
| A:PHE246>CYS | 0.67 | DESTABILIZING | 1.1 | -0.1 |
| A:TYR313>MET | 0.67 | DESTABILIZING | 1.03 | -0.06 |
| A:TYR313>PRO | 0.67 | DESTABILIZING | 1.6 | -0.06 |
| A:PHE246>ASP | 0.7 | DESTABILIZING | 0.43 | 0.7 |
| A:PHE246>GLY | 0.71 | DESTABILIZING | 1.15 | -0.15 |
| A:PHE246>SER | 0.71 | DESTABILIZING | 1.06 | -0.03 |
| A:TYR313>ASN | 0.74 | DESTABILIZING | 1.35 | 0.09 |
| A:GLU340>CYS | 0.75 | DESTABILIZING | 1.67 | -0.23 |
| A:TYR313>LYS | 0.78 | DESTABILIZING | 0.18 | 1.01 |
| A:PHE246>ALA | 0.84 | DESTABILIZING | 1.2 | 0.07 |
| A:GLU340>ALA | 0.85 | DESTABILIZING | 1.73 | -0.18 |
| A:GLU340>ARG | 0.96 | DESTABILIZING | 0.84 | 1.12 |
| A:GLU340>GLY | 0.97 | DESTABILIZING | 2 | -0.2 |
| A:TYR313>THR | 1 | DESTABILIZING | 1.37 | 0.19 |
| A:TYR313>ARG | 1.17 | DESTABILIZING | 1.48 | 0.63 |
| A:TYR313>ASP | 1.24 | DESTABILIZING | 1.69 | 0.81 |
| A:TYR313>VAL | 1.54 | DESTABILIZING | 2.95 | 0.05 |
| A:TYR313>ALA | 1.6 | DESTABILIZING | 3.46 | -0.05 |
| A:TYR313>CYS | 1.61 | DESTABILIZING | 3.56 | -0.1 |
| A:TYR313>SER | 1.89 | DESTABILIZING | 3.29 | -0.02 |
| A:TYR313>GLY | 2.2 | DESTABILIZING | 4.65 | -0.05 |
| Multipoint simultaneous mutagenesis | | | | |
| A:TYR313>GLY.A:GLU340>ARG.A:ASN396>ARG | 1.88 | DESTABILIZING | 3.19 | 0.32 |

**Part 2. Results of analysis using the 6Q6K PDB structure**

| Mutation | Mutation Energy  (kcal/mol) | | Effect of Mutation | vDW Term | Electrostatic Term |
| --- | --- | --- | --- | --- | --- |
| Alanine scanning | | | | | |
| B:GLY344>ALA | | -0.19 | NEUTRAL | -0.41 | 0.03 |
| B:ASP127>ALA | | -0.11 | NEUTRAL | 0.35 | -0.59 |
| B:CYS342>ALA | | 0 | NEUTRAL | -0.05 | -0.04 |
| B:VAL343>ALA | | 0.03 | NEUTRAL | 0.06 | 0.01 |
| B:ASN234>ALA | | 0.16 | NEUTRAL | 0.66 | -0.38 |
| B:PHE128>ALA | | 0.29 | NEUTRAL | 0.8 | -0.23 |
| B:GLU235>ALA | | 0.35 | NEUTRAL | 2.71 | -2.13 |
| B:TYR244>ALA | | 0.4 | NEUTRAL | 1.04 | -0.33 |
| A:PHE347>ALA | | 0.56 | DESTABILIZING | 1.12 | -0.01 |
| B:VAL398>ALA | | 0.58 | DESTABILIZING | 1.19 | -0.08 |
| B:ASN396>ALA | | 0.7 | DESTABILIZING | 1.64 | -0.27 |
| B:TRP179>ALA | | 0.8 | DESTABILIZING | 1.84 | -0.28 |
| B:GLU340>ALA | | 0.98 | DESTABILIZING | 1.64 | 0.23 |
| B:TRP381>ALA | | 1.06 | DESTABILIZING | 2.18 | -0.13 |
| B:PHE246>ALA | | 1.12 | DESTABILIZING | 2.2 | -0.17 |
| B:TYR313>ALA | | 1.65 | DESTABILIZING | 3.35 | -0.08 |
| Saturation mutagenesis | | | | | |
| B:PHE246>LYS | | -0.51 | STABILIZING | -0.83 | -0.23 |
| B:ASN396>GLN | | -0.5 | NEUTRAL | -1.21 | 0.09 |
| B:ASN396>ASN | | -0.43 | NEUTRAL | -0.72 | -0.14 |
| B:VAL398>ARG | | -0.36 | NEUTRAL | -1.19 | 0.42 |
| B:GLU340>PHE | | -0.35 | NEUTRAL | -0.91 | 0.23 |
| A:PHE347>PHE | | -0.26 | NEUTRAL | -0.57 | 0.04 |
| B:ASN396>TRP | | -0.26 | NEUTRAL | -0.37 | -0.01 |
| B:VAL398>LEU | | -0.25 | NEUTRAL | -0.75 | 0.23 |
| B:GLU340>GLN | | -0.21 | NEUTRAL | -0.44 | 0.02 |
| A:PHE347>LEU | | -0.18 | NEUTRAL | -0.68 | -0.01 |
| A:PHE347>TYR | | -0.17 | NEUTRAL | -0.56 | 0.1 |
| B:ASN396>LEU | | -0.17 | NEUTRAL | -0.18 | -0.17 |
| B:ASN396>MET | | -0.16 | NEUTRAL | -0.26 | -0.1 |
| B:TYR313>PHE | | -0.11 | NEUTRAL | -0.1 | -0.08 |
| B:PHE246>PHE | | -0.1 | NEUTRAL | -0.24 | 0.04 |
| B:TYR313>TYR | | -0.1 | NEUTRAL | -0.25 | 0.04 |
| B:TRP381>TRP | | -0.08 | NEUTRAL | -0.17 | 0.02 |
| B:GLU340>GLU | | -0.07 | NEUTRAL | -0.18 | 0.03 |
| B:VAL398>VAL | | -0.05 | NEUTRAL | -0.14 | 0.05 |
| B:TRP179>TRP | | -0.03 | NEUTRAL | -0.09 | 0.02 |
| B:PHE246>TYR | | -0.03 | NEUTRAL | -1.15 | 1.1 |
| B:GLU340>HIS | | 0 | NEUTRAL | -0.14 | 0.14 |
| B:VAL398>THR | | 0.01 | NEUTRAL | 0.07 | -0.06 |
| A:PHE347>HIS | | 0.02 | NEUTRAL | -0.36 | -0.04 |
| B:ASN396>ARG | | 0.03 | NEUTRAL | -0.24 | 0.18 |
| B:TRP381>GLN | | 0.04 | NEUTRAL | -0.09 | 0.12 |
| B:VAL398>GLN | | 0.06 | NEUTRAL | -0.21 | 0.28 |
| B:TYR313>HIS | | 0.07 | NEUTRAL | 0.14 | -0.11 |
| B:ASN396>ILE | | 0.07 | NEUTRAL | 0.43 | -0.25 |
| A:PHE347>THR | | 0.09 | NEUTRAL | -0.51 | 0.04 |
| B:TRP179>TYR | | 0.11 | NEUTRAL | 0.58 | -0.35 |
| B:TRP179>PHE | | 0.13 | NEUTRAL | 0.49 | -0.23 |
| B:VAL398>HIS | | 0.14 | NEUTRAL | -0.34 | 0.66 |
| B:GLU340>TYR | | 0.16 | NEUTRAL | -0.24 | 0.58 |
| B:TYR313>TRP | | 0.17 | NEUTRAL | 0.09 | 0.27 |
| B:VAL398>ILE | | 0.17 | NEUTRAL | 0.13 | 0.19 |
| B:PHE246>HIS | | 0.19 | NEUTRAL | 0.42 | -0.09 |
| B:GLU340>ILE | | 0.19 | NEUTRAL | 0.12 | 0.23 |
| A:PHE347>MET | | 0.21 | NEUTRAL | -0.3 | 0.05 |
| B:TRP381>PHE | | 0.21 | NEUTRAL | 0.51 | -0.1 |
| A:PHE347>GLU | | 0.25 | NEUTRAL | -1.23 | 0.73 |
| B:GLU340>LEU | | 0.25 | NEUTRAL | 0.24 | 0.26 |
| B:TRP381>LEU | | 0.25 | NEUTRAL | 0.62 | -0.13 |
| A:PHE347>ASP | | 0.26 | NEUTRAL | -0.4 | 0.45 |
| B:TRP179>HIS | | 0.26 | NEUTRAL | 0.8 | -0.29 |
| B:PHE246>ARG | | 0.26 | NEUTRAL | 0.73 | -0.22 |
| B:VAL398>ASN | | 0.27 | NEUTRAL | -0.12 | 0.65 |
| B:GLU340>ASP | | 0.28 | NEUTRAL | 0.16 | 0.37 |
| B:ASN396>ASP | | 0.29 | NEUTRAL | -0.25 | 0.84 |
| B:ASN396>HIS | | 0.29 | NEUTRAL | 0.97 | -0.4 |
| B:VAL398>SER | | 0.3 | NEUTRAL | 0.48 | 0.09 |
| A:PHE347>LYS | | 0.31 | NEUTRAL | -0.98 | 0.26 |
| A:PHE347>ARG | | 0.32 | NEUTRAL | -0.81 | 0.37 |
| A:PHE347>GLN | | 0.32 | NEUTRAL | -0.93 | 0.17 |
| B:PHE246>TRP | | 0.33 | NEUTRAL | -0.45 | 1.13 |
| B:GLU340>VAL | | 0.33 | NEUTRAL | 0.37 | 0.26 |
| A:PHE347>PRO | | 0.34 | NEUTRAL | 0.67 | 0 |
| B:TRP381>HIS | | 0.35 | NEUTRAL | 1 | -0.3 |
| B:VAL398>LYS | | 0.35 | NEUTRAL | -1.05 | 1.7 |
| A:PHE347>ASN | | 0.36 | NEUTRAL | 0.11 | -0.07 |
| B:TRP179>MET | | 0.36 | NEUTRAL | 1.01 | -0.28 |
| B:VAL398>PRO | | 0.37 | NEUTRAL | 0.75 | -0.06 |
| B:ASN396>PHE | | 0.38 | NEUTRAL | 1.11 | -0.24 |
| B:VAL398>CYS | | 0.38 | NEUTRAL | 0.71 | 0.07 |
| A:PHE347>VAL | | 0.39 | NEUTRAL | 0.47 | 0 |
| A:PHE347>TRP | | 0.4 | NEUTRAL | 0.48 | 0.04 |
| B:PHE246>MET | | 0.41 | NEUTRAL | 0.91 | -0.13 |
| A:PHE347>CYS | | 0.42 | NEUTRAL | 0.92 | 0.03 |
| B:TRP179>GLN | | 0.43 | NEUTRAL | 0.9 | -0.05 |
| B:PHE246>GLN | | 0.43 | NEUTRAL | 0.7 | 0.11 |
| B:ASN396>LYS | | 0.44 | NEUTRAL | -0.29 | 0.95 |
| B:ASN396>TYR | | 0.45 | NEUTRAL | 1.23 | -0.24 |
| B:TYR313>ARG | | 0.46 | NEUTRAL | -0.49 | -0.12 |
| B:TRP381>ILE | | 0.49 | NEUTRAL | 1.05 | -0.1 |
| B:VAL398>GLU | | 0.49 | NEUTRAL | -0.76 | 1.68 |
| B:TRP179>LEU | | 0.51 | DESTABILIZING | 1.2 | -0.2 |
| B:ASN396>THR | | 0.54 | DESTABILIZING | 1.31 | -0.2 |
| B:GLU340>LYS | | 0.55 | DESTABILIZING | 1.32 | -0.22 |
| B:GLU340>PRO | | 0.55 | DESTABILIZING | 0.65 | 0.37 |
| A:PHE347>ALA | | 0.56 | DESTABILIZING | 1.12 | -0.01 |
| B:TRP381>TYR | | 0.57 | DESTABILIZING | 1.03 | 0.12 |
| B:ASN396>PRO | | 0.58 | DESTABILIZING | 1.37 | -0.23 |
| B:VAL398>ALA | | 0.58 | DESTABILIZING | 1.19 | -0.08 |
| A:PHE347>SER | | 0.59 | DESTABILIZING | -0.23 | 0.1 |
| B:TRP179>GLU | | 0.59 | DESTABILIZING | 0.95 | 0.22 |
| B:ASN396>CYS | | 0.6 | DESTABILIZING | 1.5 | -0.28 |
| B:ASN396>GLU | | 0.6 | DESTABILIZING | 0.9 | 0.32 |
| B:GLU340>THR | | 0.62 | DESTABILIZING | 0.93 | 0.26 |
| B:PHE246>LEU | | 0.63 | DESTABILIZING | 1.32 | -0.14 |
| B:GLU340>ASN | | 0.64 | DESTABILIZING | 0.97 | 0.28 |
| B:TRP381>ASN | | 0.64 | DESTABILIZING | 1.34 | -0.09 |
| B:ASN396>VAL | | 0.64 | DESTABILIZING | 1.49 | -0.17 |
| B:ASN396>SER | | 0.66 | DESTABILIZING | 1.62 | -0.3 |
| B:TRP179>ASN | | 0.67 | DESTABILIZING | 1.48 | -0.15 |
| B:TRP179>ILE | | 0.67 | DESTABILIZING | 1.49 | -0.16 |
| B:TRP179>THR | | 0.67 | DESTABILIZING | 1.58 | -0.25 |
| B:TRP179>VAL | | 0.67 | DESTABILIZING | 1.56 | -0.23 |
| B:TRP381>GLU | | 0.69 | DESTABILIZING | 0.22 | 1.12 |
| B:GLU340>MET | | 0.7 | DESTABILIZING | 1.14 | 0.26 |
| B:ASN396>ALA | | 0.7 | DESTABILIZING | 1.64 | -0.27 |
| B:TRP381>LYS | | 0.71 | DESTABILIZING | 1.66 | -0.27 |
| B:TRP179>PRO | | 0.73 | DESTABILIZING | 1.72 | -0.29 |
| B:GLU340>SER | | 0.73 | DESTABILIZING | 1.1 | 0.25 |
| B:TRP179>ASP | | 0.74 | DESTABILIZING | 1.35 | 0.12 |
| B:TRP179>LYS | | 0.75 | DESTABILIZING | 1.17 | 0.33 |
| B:TRP179>CYS | | 0.76 | DESTABILIZING | 1.79 | -0.28 |
| B:TRP179>SER | | 0.78 | DESTABILIZING | 1.79 | -0.27 |
| B:PHE246>GLU | | 0.78 | DESTABILIZING | 0.62 | 0.88 |
| B:TYR313>LEU | | 0.78 | DESTABILIZING | 1.38 | -0.09 |
| B:TRP381>THR | | 0.78 | DESTABILIZING | 1.65 | -0.14 |
| B:VAL398>GLY | | 0.78 | DESTABILIZING | 1.62 | -0.11 |
| B:TRP179>ALA | | 0.8 | DESTABILIZING | 1.84 | -0.28 |
| B:PHE246>ILE | | 0.8 | DESTABILIZING | 1.61 | -0.1 |
| B:TRP179>GLY | | 0.81 | DESTABILIZING | 1.88 | -0.29 |
| B:TRP381>VAL | | 0.81 | DESTABILIZING | 1.74 | -0.15 |
| B:ASN396>GLY | | 0.82 | DESTABILIZING | 1.87 | -0.3 |
| A:PHE347>GLY | | 0.85 | DESTABILIZING | 1.72 | -0.01 |
| B:GLU340>CYS | | 0.88 | DESTABILIZING | 1.52 | 0.19 |
| B:PHE246>VAL | | 0.89 | DESTABILIZING | 1.82 | -0.16 |
| B:TYR313>LYS | | 0.9 | DESTABILIZING | -0.11 | 0.36 |
| B:TRP381>ASP | | 0.9 | DESTABILIZING | 1.4 | 0.37 |
| B:PHE246>PRO | | 0.91 | DESTABILIZING | 1.87 | -0.21 |
| B:PHE246>THR | | 0.91 | DESTABILIZING | 1.83 | -0.18 |
| B:TRP381>SER | | 0.91 | DESTABILIZING | 1.91 | -0.15 |
| B:PHE246>ASN | | 0.93 | DESTABILIZING | 1.77 | -0.03 |
| B:TRP381>CYS | | 0.93 | DESTABILIZING | 1.99 | -0.16 |
| B:PHE246>CYS | | 0.94 | DESTABILIZING | 1.9 | -0.16 |
| B:TRP381>PRO | | 0.94 | DESTABILIZING | 2.09 | -0.26 |
| B:TYR313>GLN | | 0.97 | DESTABILIZING | 0.04 | 0.08 |
| B:GLU340>GLY | | 0.97 | DESTABILIZING | 1.64 | 0.21 |
| A:PHE347>ILE | | 0.98 | DESTABILIZING | 1.48 | 0.05 |
| B:GLU340>ALA | | 0.98 | DESTABILIZING | 1.64 | 0.23 |
| B:PHE246>SER | | 1.05 | DESTABILIZING | 2.07 | -0.16 |
| B:TRP381>ALA | | 1.06 | DESTABILIZING | 2.18 | -0.13 |
| B:TYR313>MET | | 1.07 | DESTABILIZING | 1.77 | -0.15 |
| B:PHE246>ALA | | 1.12 | DESTABILIZING | 2.2 | -0.17 |
| B:PHE246>ASP | | 1.12 | DESTABILIZING | 1.74 | 0.39 |
| B:VAL398>PHE | | 1.12 | DESTABILIZING | 2.17 | 0.05 |
| B:PHE246>GLY | | 1.15 | DESTABILIZING | 2.27 | -0.2 |
| B:TRP179>ARG | | 1.16 | DESTABILIZING | 2.52 | -0.21 |
| B:TRP381>GLY | | 1.16 | DESTABILIZING | 2.33 | -0.1 |
| B:TRP381>MET | | 1.18 | DESTABILIZING | 2.36 | -0.03 |
| B:TYR313>PRO | | 1.29 | DESTABILIZING | 2.7 | -0.14 |
| B:VAL398>TYR | | 1.33 | DESTABILIZING | 2.29 | 0.44 |
| B:TYR313>ILE | | 1.34 | DESTABILIZING | 2.28 | -0.07 |
| B:TYR313>ASN | | 1.35 | DESTABILIZING | 2.51 | -0.1 |
| B:GLU340>TRP | | 1.36 | DESTABILIZING | 2.15 | 0.55 |
| B:TYR313>ASP | | 1.45 | DESTABILIZING | 2.35 | 0.3 |
| B:VAL398>ASP | | 1.47 | DESTABILIZING | 0.37 | 2.57 |
| B:TRP381>ARG | | 1.55 | DESTABILIZING | 2.06 | 1 |
| B:TYR313>ALA | | 1.65 | DESTABILIZING | 3.35 | -0.08 |
| B:TYR313>CYS | | 1.66 | DESTABILIZING | 3.36 | -0.06 |
| B:VAL398>MET | | 1.75 | DESTABILIZING | 3.19 | 0.32 |
| B:TYR313>SER | | 1.95 | DESTABILIZING | 2.47 | 0.07 |
| B:TYR313>VAL | | 1.97 | DESTABILIZING | 3.74 | -0.13 |
| B:TYR313>GLY | | 2.07 | DESTABILIZING | 4.17 | -0.13 |
| B:TYR313>GLU | | 2.11 | DESTABILIZING | 1.39 | 1.35 |
| B:TYR313>THR | | 2.19 | DESTABILIZING | 3.65 | -0.15 |
| B:VAL398>TRP | | 2.78 | DESTABILIZING | 4.11 | 0.58 |
| B:GLU340>ARG | | 3.29 | DESTABILIZING | 4.77 | 1.78 |
| Multipoint simultaneous mutagenesis | | | | | |
| B:TYR313>GLY.B:GLU340>ARG.B:ASN396>ARG | | 5.83 | DESTABILIZING | 9.66 | 1.13 |

**Part 3. Results of analysis using the 6Q6L PDB structure**

| Mutation | Mutation Energy  (kcal/mol) | | Effect of Mutation | vDW Term | Electrostatic Term |
| --- | --- | --- | --- | --- | --- |
| Alanine scanning | | | | | |
| A:PHE128>ALA | | 0.22 | NEUTRAL | 0.64 | -0.21 |
| A:GLU340>ALA | | 0.49 | NEUTRAL | 1.01 | -0.1 |
| A:VAL398>ALA | | 0.51 | DESTABILIZING | 1.02 | -0.04 |
| A:ASN396>ALA | | 0.62 | DESTABILIZING | 1.46 | -0.2 |
| A:PHE397>ALA | | 0.74 | DESTABILIZING | 1.27 | 0.01 |
| B:PHE316>ALA | | 0.74 | DESTABILIZING | 1.51 | 0 |
| A:TRP381>ALA | | 0.75 | DESTABILIZING | 1.68 | -0.25 |
| A:LEU314>ALA | | 1.07 | DESTABILIZING | 1.95 | 0.01 |
| A:PHE246>ALA | | 1.09 | DESTABILIZING | 2.15 | -0.13 |
| B:PHE347>ALA | | 1.11 | DESTABILIZING | 2.25 | 0.01 |
| A:TYR313>ALA | | 2.17 | DESTABILIZING | 4.3 | 0.07 |
| Saturation mutagenesis | | | | | |
| A:LEU314>TRP | | -0.46 | NEUTRAL | -0.97 | 0.09 |
| B:PHE347>TRP | | -0.42 | NEUTRAL | -0.92 | 0.06 |
| A:PHE246>LYS | | -0.33 | NEUTRAL | -0.79 | 0.02 |
| A:VAL398>ARG | | -0.23 | NEUTRAL | -0.23 | -0.24 |
| A:LEU314>TYR | | -0.2 | NEUTRAL | -0.68 | 0.24 |
| A:VAL398>GLN | | -0.19 | NEUTRAL | -0.53 | 0.17 |
| B:PHE316>TRP | | -0.17 | NEUTRAL | -0.55 | 0.03 |
| A:LEU314>LEU | | -0.15 | NEUTRAL | -0.33 | 0.04 |
| A:VAL398>ILE | | -0.12 | NEUTRAL | -0.28 | 0.05 |
| A:VAL398>LEU | | -0.12 | NEUTRAL | -0.23 | -0.02 |
| A:ASN396>GLN | | -0.1 | NEUTRAL | -0.41 | 0.21 |
| A:VAL398>ASN | | -0.1 | NEUTRAL | -0.64 | 0.49 |
| B:PHE316>TYR | | -0.07 | NEUTRAL | -0.47 | 0.11 |
| A:PHE397>TYR | | -0.03 | NEUTRAL | -0.05 | 0 |
| B:PHE347>TYR | | -0.03 | NEUTRAL | -0.2 | 0.1 |
| A:ASN396>LYS | | -0.02 | NEUTRAL | 1.03 | -1.08 |
| A:PHE246>PHE | | -0.01 | NEUTRAL | -0.02 | 0 |
| A:TYR313>TYR | | -0.01 | NEUTRAL | -0.01 | 0 |
| A:TRP381>TRP | | -0.01 | NEUTRAL | -0.01 | 0 |
| A:ASN396>ASN | | -0.01 | NEUTRAL | -0.01 | 0 |
| A:PHE397>PHE | | -0.01 | NEUTRAL | -0.01 | 0 |
| A:VAL398>VAL | | -0.01 | NEUTRAL | -0.01 | 0 |
| B:PHE347>PHE | | -0.01 | NEUTRAL | 0 | -0.01 |
| B:PHE316>PHE | | 0 | NEUTRAL | 0 | 0 |
| A:TRP381>LYS | | 0.05 | NEUTRAL | 0.51 | -0.41 |
| A:TRP381>TYR | | 0.05 | NEUTRAL | 0.2 | -0.11 |
| A:TRP381>PHE | | 0.06 | NEUTRAL | 0.23 | -0.11 |
| A:TYR313>TRP | | 0.08 | NEUTRAL | 0.13 | 0.01 |
| A:VAL398>THR | | 0.12 | NEUTRAL | 0.28 | -0.02 |
| A:ASN396>ARG | | 0.15 | NEUTRAL | 0.94 | -0.62 |
| A:TRP381>GLN | | 0.16 | NEUTRAL | -0.11 | 0.4 |
| A:PHE397>HIS | | 0.16 | NEUTRAL | 0.27 | 0 |
| A:LEU314>ARG | | 0.18 | NEUTRAL | 0.07 | 0.11 |
| A:TYR313>PHE | | 0.2 | NEUTRAL | 0.48 | -0.03 |
| A:TRP381>HIS | | 0.2 | NEUTRAL | 0.58 | -0.17 |
| A:VAL398>PRO | | 0.2 | NEUTRAL | 0.45 | -0.06 |
| A:ASN396>LEU | | 0.21 | NEUTRAL | 0.5 | 0 |
| B:PHE347>ARG | | 0.21 | NEUTRAL | 0.08 | 0.11 |
| A:LEU314>MET | | 0.26 | NEUTRAL | 0.16 | 0.03 |
| A:LEU314>PHE | | 0.26 | NEUTRAL | 0.61 | -0.01 |
| A:TRP381>LEU | | 0.26 | NEUTRAL | 0.72 | -0.22 |
| A:VAL398>SER | | 0.28 | NEUTRAL | 0.54 | 0 |
| B:PHE316>HIS | | 0.28 | NEUTRAL | 0.43 | 0.03 |
| A:LEU314>ASN | | 0.31 | NEUTRAL | 0.54 | 0.06 |
| A:LEU314>HIS | | 0.31 | NEUTRAL | 0.59 | 0.07 |
| A:TRP381>ILE | | 0.31 | NEUTRAL | 0.83 | -0.22 |
| A:PHE397>ASN | | 0.31 | NEUTRAL | 0.38 | 0.11 |
| A:VAL398>GLU | | 0.31 | NEUTRAL | -0.16 | 0.76 |
| A:LEU314>GLU | | 0.32 | NEUTRAL | -0.26 | 0.63 |
| A:PHE397>LEU | | 0.32 | NEUTRAL | 0.57 | 0 |
| B:PHE347>HIS | | 0.33 | NEUTRAL | 0.44 | 0.05 |
| A:PHE246>GLN | | 0.34 | NEUTRAL | 0.47 | 0.11 |
| A:PHE246>TRP | | 0.34 | NEUTRAL | 0.39 | 0.31 |
| B:PHE316>LEU | | 0.34 | NEUTRAL | 0.72 | 0 |
| A:PHE246>ARG | | 0.35 | NEUTRAL | 0.85 | -0.25 |
| A:PHE246>HIS | | 0.35 | NEUTRAL | 0.69 | -0.03 |
| A:VAL398>CYS | | 0.35 | NEUTRAL | 0.74 | 0 |
| A:LEU314>LYS | | 0.36 | NEUTRAL | -0.44 | 0.48 |
| A:TRP381>GLU | | 0.36 | NEUTRAL | -0.23 | 0.91 |
| A:PHE246>MET | | 0.38 | NEUTRAL | 0.71 | -0.01 |
| A:ASN396>MET | | 0.38 | NEUTRAL | 0.75 | 0 |
| A:LEU314>ILE | | 0.39 | NEUTRAL | 0.72 | 0.03 |
| A:ASN396>VAL | | 0.39 | NEUTRAL | 0.94 | -0.14 |
| B:PHE347>LYS | | 0.41 | NEUTRAL | -0.18 | 0.52 |
| A:ASN396>GLU | | 0.42 | NEUTRAL | -0.91 | 1.75 |
| A:TRP381>ASN | | 0.46 | NEUTRAL | 1.08 | -0.18 |
| B:PHE347>LEU | | 0.46 | NEUTRAL | 0.77 | 0 |
| A:ASN396>THR | | 0.47 | NEUTRAL | 1.15 | -0.2 |
| A:ASN396>ILE | | 0.48 | NEUTRAL | 1.23 | -0.24 |
| A:VAL398>ASP | | 0.48 | NEUTRAL | 0.66 | 0.27 |
| A:LEU314>GLN | | 0.51 | DESTABILIZING | 0.38 | 0.22 |
| A:PHE397>ASP | | 0.51 | DESTABILIZING | 0.41 | 0.49 |
| A:VAL398>ALA | | 0.51 | DESTABILIZING | 1.02 | -0.04 |
| B:PHE316>ILE | | 0.51 | DESTABILIZING | 1.06 | 0 |
| A:TRP381>VAL | | 0.54 | DESTABILIZING | 1.29 | -0.25 |
| A:ASN396>SER | | 0.54 | DESTABILIZING | 1.27 | -0.19 |
| B:PHE316>ASN | | 0.54 | DESTABILIZING | 1.07 | 0.02 |
| B:PHE316>VAL | | 0.54 | DESTABILIZING | 1.14 | 0 |
| B:PHE347>MET | | 0.54 | DESTABILIZING | 0.76 | -0.01 |
| A:LEU314>ASP | | 0.55 | DESTABILIZING | 0.67 | 0.42 |
| A:TRP381>THR | | 0.55 | DESTABILIZING | 1.31 | -0.25 |
| A:PHE397>MET | | 0.55 | DESTABILIZING | 0.98 | 0 |
| A:TRP381>ARG | | 0.56 | DESTABILIZING | 1.44 | -0.32 |
| B:PHE316>MET | | 0.58 | DESTABILIZING | 0.87 | 0 |
| A:ASN396>PRO | | 0.6 | DESTABILIZING | 1.42 | -0.2 |
| A:PHE397>CYS | | 0.6 | DESTABILIZING | 1.14 | 0 |
| A:PHE397>THR | | 0.6 | DESTABILIZING | 0.84 | 0.04 |
| A:LEU314>VAL | | 0.61 | DESTABILIZING | 1.12 | 0.03 |
| A:ASN396>ALA | | 0.62 | DESTABILIZING | 1.46 | -0.2 |
| B:PHE316>ASP | | 0.62 | DESTABILIZING | 1.13 | 0.15 |
| B:PHE316>GLN | | 0.62 | DESTABILIZING | 1.06 | 0.03 |
| B:PHE316>PRO | | 0.62 | DESTABILIZING | 1.28 | 0 |
| A:PHE246>LEU | | 0.64 | DESTABILIZING | 1.28 | -0.08 |
| A:TRP381>PRO | | 0.64 | DESTABILIZING | 1.52 | -0.28 |
| A:ASN396>CYS | | 0.64 | DESTABILIZING | 1.32 | 0.01 |
| A:TYR313>HIS | | 0.65 | DESTABILIZING | 1.23 | -0.01 |
| B:PHE316>GLU | | 0.65 | DESTABILIZING | 1.09 | 0.09 |
| B:PHE347>PRO | | 0.65 | DESTABILIZING | 1.37 | 0.01 |
| B:PHE316>CYS | | 0.66 | DESTABILIZING | 1.37 | 0.01 |
| A:TRP381>ASP | | 0.68 | DESTABILIZING | 1.09 | 0.23 |
| A:TRP381>CYS | | 0.69 | DESTABILIZING | 1.57 | -0.23 |
| B:PHE316>THR | | 0.69 | DESTABILIZING | 1.33 | 0.01 |
| A:VAL398>GLY | | 0.7 | DESTABILIZING | 1.35 | -0.05 |
| A:ASN396>ASP | | 0.71 | DESTABILIZING | 1.33 | 0.08 |
| A:ASN396>HIS | | 0.71 | DESTABILIZING | 1.6 | -0.08 |
| A:PHE246>TYR | | 0.72 | DESTABILIZING | 0.75 | 0.69 |
| A:TRP381>SER | | 0.72 | DESTABILIZING | 1.57 | -0.23 |
| B:PHE316>ARG | | 0.72 | DESTABILIZING | 0.74 | 0.06 |
| B:PHE316>LYS | | 0.72 | DESTABILIZING | 1.39 | 0.06 |
| A:PHE246>ILE | | 0.73 | DESTABILIZING | 1.42 | -0.08 |
| B:PHE347>ASN | | 0.73 | DESTABILIZING | 1.27 | 0.02 |
| A:PHE397>ALA | | 0.74 | DESTABILIZING | 1.27 | 0.01 |
| B:PHE316>ALA | | 0.74 | DESTABILIZING | 1.51 | 0 |
| A:PHE246>GLU | | 0.75 | DESTABILIZING | 0.56 | 0.85 |
| A:TRP381>ALA | | 0.75 | DESTABILIZING | 1.68 | -0.25 |
| B:PHE316>SER | | 0.76 | DESTABILIZING | 1.4 | 0.02 |
| B:PHE316>GLY | | 0.77 | DESTABILIZING | 1.57 | 0.01 |
| A:LEU314>THR | | 0.78 | DESTABILIZING | 1.41 | 0.04 |
| A:ASN396>GLY | | 0.78 | DESTABILIZING | 1.78 | -0.22 |
| A:VAL398>MET | | 0.79 | DESTABILIZING | 1.38 | 0.23 |
| A:PHE397>SER | | 0.8 | DESTABILIZING | 0.94 | 0.06 |
| A:TRP381>GLY | | 0.81 | DESTABILIZING | 1.8 | -0.26 |
| A:LEU314>PRO | | 0.82 | DESTABILIZING | 1.53 | 0.02 |
| A:PHE397>LYS | | 0.83 | DESTABILIZING | 0.96 | 0.42 |
| B:PHE347>ILE | | 0.84 | DESTABILIZING | 1.35 | 0 |
| A:LEU314>CYS | | 0.85 | DESTABILIZING | 1.7 | 0.01 |
| B:PHE347>GLN | | 0.87 | DESTABILIZING | 1.08 | 0.1 |
| A:PHE246>ASN | | 0.88 | DESTABILIZING | 1.66 | 0.01 |
| A:PHE246>PRO | | 0.89 | DESTABILIZING | 1.82 | -0.16 |
| A:PHE246>VAL | | 0.89 | DESTABILIZING | 1.77 | -0.1 |
| B:PHE347>ASP | | 0.91 | DESTABILIZING | 1.38 | 0.32 |
| A:LEU314>SER | | 0.92 | DESTABILIZING | 1.7 | 0.02 |
| B:PHE347>GLU | | 0.93 | DESTABILIZING | 0.7 | 0.59 |
| B:PHE347>VAL | | 0.96 | DESTABILIZING | 1.79 | 0.01 |
| A:PHE246>THR | | 0.98 | DESTABILIZING | 1.96 | -0.13 |
| A:TYR313>GLN | | 0.98 | DESTABILIZING | 0.92 | 0.83 |
| A:ASN396>PHE | | 0.98 | DESTABILIZING | 2.04 | 0.09 |
| B:PHE347>CYS | | 1.02 | DESTABILIZING | 2.11 | 0.01 |
| A:TYR313>GLU | | 1.03 | DESTABILIZING | -0.15 | 1.62 |
| A:PHE397>GLY | | 1.03 | DESTABILIZING | 1.76 | 0.02 |
| A:PHE246>CYS | | 1.05 | DESTABILIZING | 2.08 | -0.1 |
| A:PHE246>SER | | 1.06 | DESTABILIZING | 2.1 | -0.11 |
| A:LEU314>ALA | | 1.07 | DESTABILIZING | 1.95 | 0.01 |
| A:PHE246>ASP | | 1.08 | DESTABILIZING | 1.71 | 0.36 |
| A:PHE246>ALA | | 1.09 | DESTABILIZING | 2.15 | -0.13 |
| B:PHE347>ALA | | 1.11 | DESTABILIZING | 2.25 | 0.01 |
| A:PHE246>GLY | | 1.14 | DESTABILIZING | 2.22 | -0.12 |
| A:TYR313>LEU | | 1.14 | DESTABILIZING | 2.24 | 0.09 |
| A:VAL398>TRP | | 1.15 | DESTABILIZING | 1.68 | 0.69 |
| B:PHE347>THR | | 1.15 | DESTABILIZING | 1.94 | 0.03 |
| B:PHE347>SER | | 1.16 | DESTABILIZING | 2.09 | 0.03 |
| A:LEU314>GLY | | 1.18 | DESTABILIZING | 2.11 | 0.02 |
| B:PHE347>GLY | | 1.19 | DESTABILIZING | 2.35 | 0 |
| A:PHE397>ARG | | 1.2 | DESTABILIZING | 0.84 | 0.28 |
| A:PHE397>VAL | | 1.26 | DESTABILIZING | 2.29 | 0 |
| A:TYR313>ASN | | 1.3 | DESTABILIZING | 2.45 | 0.09 |
| A:TYR313>ILE | | 1.33 | DESTABILIZING | 2.47 | 0.23 |
| A:PHE397>GLN | | 1.39 | DESTABILIZING | 1.55 | 0.07 |
| A:TRP381>MET | | 1.45 | DESTABILIZING | 3.02 | -0.14 |
| A:PHE397>GLU | | 1.52 | DESTABILIZING | 1.55 | 0.55 |
| A:TYR313>ASP | | 1.54 | DESTABILIZING | 2.58 | 0.48 |
| A:ASN396>TYR | | 1.57 | DESTABILIZING | 3.32 | -0.03 |
| A:PHE397>PRO | | 1.57 | DESTABILIZING | 3.21 | -0.05 |
| A:VAL398>TYR | | 1.59 | DESTABILIZING | 2.71 | 0.59 |
| A:TYR313>THR | | 1.61 | DESTABILIZING | 2.98 | 0.12 |
| A:VAL398>HIS | | 1.61 | DESTABILIZING | 2.74 | 0.52 |
| A:TYR313>VAL | | 1.63 | DESTABILIZING | 3.25 | 0.05 |
| A:TYR313>MET | | 1.65 | DESTABILIZING | 2.87 | 0.42 |
| A:TYR313>PRO | | 1.86 | DESTABILIZING | 3.73 | 0.03 |
| A:VAL398>LYS | | 1.86 | DESTABILIZING | 2.44 | 1.31 |
| A:VAL398>PHE | | 1.92 | DESTABILIZING | 3.37 | 0.55 |
| A:TYR313>LYS | | 2.03 | DESTABILIZING | 2.5 | 1.42 |
| A:TYR313>SER | | 2.04 | DESTABILIZING | 3.78 | 0.15 |
| A:TYR313>CYS | | 2.13 | DESTABILIZING | 4.23 | 0.07 |
| A:TYR313>ALA | | 2.17 | DESTABILIZING | 4.3 | 0.07 |
| A:PHE397>ILE | | 2.26 | DESTABILIZING | 4.3 | -0.02 |
| A:TYR313>GLY | | 2.45 | DESTABILIZING | 4.8 | 0.07 |
| A:PHE397>TRP | | 2.52 | DESTABILIZING | 4.29 | 0.5 |
| A:TYR313>ARG | | 2.54 | DESTABILIZING | 4.21 | 0.86 |
| A:ASN396>TRP | | 3.99 | DESTABILIZING | 7.77 | 0.37 |
| Multipoint simultaneous mutagenesis | | | | | |
| A:TYR313>GLY.A:GLU340>ARG.A:ASN396>ARG | | 1.28 | DESTABILIZING | 3.38 | -0.81 |

**Part 4. Results of analysis using the 6MOZ PDB structure**

| Mutation | Mutation Energy  (kcal/mol) | | Effect of Mutation | vDW Term | Electrostatic Term |
| --- | --- | --- | --- | --- | --- |
| Alanine scanning | | | | | |
| A:ALA238>ALA | | -0.03 | NEUTRAL | -0.08 | 0.03 |
| A:ASP127>ALA | | 0.01 | NEUTRAL | 1.34 | -1.34 |
| B:ASP315>ALA | | 0.09 | NEUTRAL | 0.65 | -0.3 |
| A:ASN234>ALA | | 0.11 | NEUTRAL | 0.73 | -0.52 |
| A:LYS346>ALA | | 0.15 | NEUTRAL | 0.84 | -0.01 |
| A:GLU340>ALA | | 0.17 | NEUTRAL | 1.28 | -0.97 |
| A:SER345>ALA | | 0.19 | NEUTRAL | 0.44 | -0.04 |
| A:PHE128>ALA | | 0.23 | NEUTRAL | 0.57 | -0.12 |
| B:PHE316>ALA | | 0.55 | DESTABILIZING | 1.19 | 0 |
| A:TRP179>ALA | | 0.76 | DESTABILIZING | 2.07 | -0.59 |
| A:LEU314>ALA | | 0.77 | DESTABILIZING | 1.32 | 0.02 |
| A:ASN396>ALA | | 0.77 | DESTABILIZING | 1.66 | -0.16 |
| A:TRP381>ALA | | 0.89 | DESTABILIZING | 2.22 | -0.48 |
| A:GLN284>ALA | | 1.11 | DESTABILIZING | 2.37 | -0.19 |
| A:TYR244>ALA | | 1.3 | DESTABILIZING | 2.71 | -0.24 |
| A:PHE246>ALA | | 1.43 | DESTABILIZING | 2.66 | -0.02 |
| A:GLU235>ALA | | 1.61 | DESTABILIZING | 4.01 | -0.84 |
| A:TYR313>ALA | | 1.77 | DESTABILIZING | 3.55 | -0.22 |
| Saturation mutagenesis | | | | | |
| A:PHE246>TRP | | -0.55 | STABILIZING | -1.48 | 0.38 |
| A:GLU235>GLN | | -0.44 | NEUTRAL | -0.2 | -0.69 |
| A:PHE246>TYR | | -0.39 | NEUTRAL | -1.18 | 0.4 |
| A:TYR244>TRP | | -0.33 | NEUTRAL | -0.7 | 0.06 |
| A:TYR313>TRP | | -0.33 | NEUTRAL | -0.99 | 0.42 |
| A:TYR244>TYR | | -0.32 | NEUTRAL | -0.95 | 0.33 |
| A:LEU314>TYR | | -0.28 | NEUTRAL | -0.3 | 0.03 |
| A:TRP381>LEU | | -0.2 | NEUTRAL | -0.03 | -0.36 |
| A:LEU314>PHE | | -0.16 | NEUTRAL | -0.21 | 0.04 |
| A:PHE316>ARG | | -0.16 | NEUTRAL | -0.09 | 0.02 |
| A:ASN396>ASN | | -0.15 | NEUTRAL | -0.51 | 0.22 |
| A:GLU235>GLU | | -0.14 | NEUTRAL | -0.43 | 0.16 |
| A:PHE316>LYS | | -0.12 | NEUTRAL | 0.04 | 0.05 |
| A:LEU314>GLN | | -0.06 | NEUTRAL | -0.27 | 0.15 |
| A:LEU314>ILE | | -0.06 | NEUTRAL | -0.11 | 0 |
| A:PHE316>TRP | | -0.06 | NEUTRAL | -0.14 | -0.01 |
| A:LEU314>TRP | | -0.05 | NEUTRAL | 0.2 | 0.04 |
| A:PHE316>GLN | | -0.04 | NEUTRAL | 0.12 | 0.01 |
| A:PHE316>MET | | -0.04 | NEUTRAL | 0.06 | 0 |
| A:TYR244>ARG | | -0.03 | NEUTRAL | -0.31 | 0.2 |
| A:PHE246>PHE | | -0.03 | NEUTRAL | -0.18 | 0.1 |
| A:PHE316>GLU | | -0.01 | NEUTRAL | 0.13 | 0.02 |
| A:PHE316>HIS | | -0.01 | NEUTRAL | 0 | 0.01 |
| A:TRP179>TRP | | 0 | NEUTRAL | 0 | 0 |
| A:GLN284>GLN | | 0 | NEUTRAL | 0 | 0 |
| A:TYR313>TYR | | 0 | NEUTRAL | 0 | 0 |
| A:LEU314>LEU | | 0 | NEUTRAL | 0 | 0 |
| A:PHE316>PHE | | 0 | NEUTRAL | 0 | 0 |
| A:PHE316>TYR | | 0 | NEUTRAL | -0.08 | 0.04 |
| A:PHE316>ILE | | 0.01 | NEUTRAL | 0.15 | 0.01 |
| A:PHE316>LEU | | 0.01 | NEUTRAL | 0.1 | 0 |
| A:TRP381>GLN | | 0.02 | NEUTRAL | 0.25 | -0.25 |
| A:TRP381>TRP | | 0.03 | NEUTRAL | -0.11 | 0.15 |
| A:TRP381>PHE | | 0.07 | NEUTRAL | 0.45 | -0.32 |
| A:PHE316>ASN | | 0.08 | NEUTRAL | 0.11 | 0.01 |
| A:PHE316>PRO | | 0.08 | NEUTRAL | 0.14 | -0.01 |
| A:PHE316>VAL | | 0.09 | NEUTRAL | 0.18 | 0.01 |
| A:PHE316>THR | | 0.1 | NEUTRAL | 0.19 | 0.01 |
| A:PHE316>ASP | | 0.11 | NEUTRAL | 0.16 | 0.01 |
| A:PHE246>LYS | | 0.12 | NEUTRAL | -0.65 | 0.82 |
| A:LEU314>ASN | | 0.12 | NEUTRAL | 0.09 | 0.11 |
| A:ASN396>HIS | | 0.12 | NEUTRAL | 0.32 | -0.12 |
| A:LEU314>ARG | | 0.13 | NEUTRAL | 0.08 | 0.39 |
| A:PHE316>CYS | | 0.13 | NEUTRAL | 0.2 | 0.01 |
| A:PHE316>SER | | 0.15 | NEUTRAL | 0.2 | 0.01 |
| A:LEU314>GLU | | 0.16 | NEUTRAL | -0.12 | 0.42 |
| A:PHE316>ALA | | 0.17 | NEUTRAL | 0.21 | 0.01 |
| A:PHE316>GLY | | 0.17 | NEUTRAL | 0.21 | 0.01 |
| A:TRP381>ILE | | 0.2 | NEUTRAL | 0.51 | -0.13 |
| A:TRP381>TYR | | 0.2 | NEUTRAL | 0.47 | -0.08 |
| A:GLU235>TYR | | 0.21 | NEUTRAL | 0.96 | -0.49 |
| A:LEU314>MET | | 0.21 | NEUTRAL | 0.51 | -0.03 |
| A:LEU314>ASP | | 0.22 | NEUTRAL | -0.21 | 0.61 |
| A:TYR313>PHE | | 0.23 | NEUTRAL | 0.45 | 0.03 |
| A:ASN396>LEU | | 0.24 | NEUTRAL | 0.38 | -0.02 |
| A:ASN396>TYR | | 0.24 | NEUTRAL | 0.63 | -0.17 |
| A:PHE246>MET | | 0.25 | NEUTRAL | 0.38 | 0.07 |
| A:ASN396>LYS | | 0.26 | NEUTRAL | 0.1 | 0.25 |
| A:ASN396>MET | | 0.26 | NEUTRAL | 0.45 | -0.09 |
| A:LEU314>VAL | | 0.27 | NEUTRAL | 0.45 | 0.01 |
| A:ASN396>PHE | | 0.27 | NEUTRAL | 0.72 | -0.17 |
| A:TYR313>MET | | 0.28 | NEUTRAL | 0.55 | -0.05 |
| A:TRP381>HIS | | 0.28 | NEUTRAL | 0.89 | -0.33 |
| A:GLU235>ARG | | 0.29 | NEUTRAL | 0.4 | 0.17 |
| A:ASN396>GLU | | 0.29 | NEUTRAL | -0.17 | 0.55 |
| A:LEU314>LYS | | 0.3 | NEUTRAL | 0.32 | 0.33 |
| A:ASN396>ILE | | 0.3 | NEUTRAL | 0.38 | 0.16 |
| A:TRP179>LYS | | 0.33 | NEUTRAL | 1.77 | -1.12 |
| A:TRP179>PHE | | 0.33 | NEUTRAL | 0.82 | -0.15 |
| A:GLN284>ILE | | 0.33 | NEUTRAL | 0.92 | -0.12 |
| A:GLN284>LEU | | 0.34 | NEUTRAL | 0.99 | -0.16 |
| A:GLU235>MET | | 0.35 | NEUTRAL | 1.51 | -0.81 |
| A:TRP179>TYR | | 0.36 | NEUTRAL | 0.2 | 0.53 |
| A:GLN284>GLU | | 0.37 | NEUTRAL | -0.12 | 0.9 |
| A:LEU314>THR | | 0.37 | NEUTRAL | 0.65 | -0.01 |
| A:TRP179>MET | | 0.4 | NEUTRAL | 1.18 | -0.37 |
| A:TYR244>PHE | | 0.4 | NEUTRAL | 0.89 | -0.1 |
| A:TYR313>LEU | | 0.4 | NEUTRAL | 0.86 | -0.05 |
| A:LEU314>HIS | | 0.4 | NEUTRAL | 0.87 | -0.02 |
| A:TRP179>HIS | | 0.41 | NEUTRAL | 1.16 | -0.33 |
| A:GLU235>LEU | | 0.42 | NEUTRAL | 1.57 | -0.72 |
| A:TYR313>GLN | | 0.42 | NEUTRAL | 0.29 | 0.43 |
| A:TRP381>LYS | | 0.42 | NEUTRAL | 1.55 | -0.7 |
| A:TYR313>ARG | | 0.45 | NEUTRAL | 0.37 | 0.47 |
| A:PHE246>HIS | | 0.48 | NEUTRAL | 0.65 | 0.25 |
| A:GLN284>PHE | | 0.48 | NEUTRAL | 1.24 | -0.1 |
| A:ASN396>TRP | | 0.48 | NEUTRAL | 0.79 | 0.19 |
| A:LEU314>PRO | | 0.49 | NEUTRAL | 0.9 | -0.05 |
| A:ASN396>GLN | | 0.49 | NEUTRAL | 0.43 | 0.52 |
| A:TRP179>LEU | | 0.5 | NEUTRAL | 1.42 | -0.44 |
| A:TYR313>HIS | | 0.51 | DESTABILIZING | 0.99 | -0.01 |
| A:GLU235>ILE | | 0.52 | DESTABILIZING | 1.76 | -0.74 |
| A:TRP381>MET | | 0.54 | DESTABILIZING | 1.52 | -0.41 |
| A:TRP381>VAL | | 0.54 | DESTABILIZING | 1.48 | -0.43 |
| A:TRP179>ARG | | 0.55 | DESTABILIZING | 2.11 | -1.02 |
| A:GLU235>HIS | | 0.57 | DESTABILIZING | 1.98 | -0.83 |
| A:GLN284>PRO | | 0.58 | DESTABILIZING | 1.34 | -0.18 |
| A:TYR313>LYS | | 0.58 | DESTABILIZING | -0.07 | 1.04 |
| A:ASN396>VAL | | 0.58 | DESTABILIZING | 0.91 | 0.17 |
| A:LEU314>CYS | | 0.59 | DESTABILIZING | 1.08 | 0.01 |
| A:GLU235>PHE | | 0.6 | DESTABILIZING | 1.81 | -0.57 |
| A:GLN284>HIS | | 0.6 | DESTABILIZING | 1.47 | -0.15 |
| A:ASN396>CYS | | 0.6 | DESTABILIZING | 1.35 | -0.09 |
| A:TRP179>ILE | | 0.62 | DESTABILIZING | 1.77 | -0.54 |
| A:GLN284>VAL | | 0.63 | DESTABILIZING | 1.5 | -0.2 |
| A:ASN396>THR | | 0.64 | DESTABILIZING | 1.14 | -0.06 |
| A:ASN396>ASP | | 0.65 | DESTABILIZING | 1.01 | 0.19 |
| A:TRP381>ASN | | 0.66 | DESTABILIZING | 1.57 | -0.29 |
| A:TRP381>GLU | | 0.66 | DESTABILIZING | 0.7 | 0.59 |
| A:TRP179>VAL | | 0.67 | DESTABILIZING | 1.89 | -0.56 |
| A:GLN284>LYS | | 0.67 | DESTABILIZING | 1.42 | 0.02 |
| A:LEU314>SER | | 0.67 | DESTABILIZING | 1.11 | 0.04 |
| A:ASN396>ARG | | 0.67 | DESTABILIZING | 0.84 | 0.38 |
| A:TYR244>LYS | | 0.68 | DESTABILIZING | 1.39 | -0.12 |
| A:ASN396>PRO | | 0.68 | DESTABILIZING | 1.37 | -0.04 |
| A:TRP179>ASN | | 0.69 | DESTABILIZING | 1.73 | -0.37 |
| A:TRP179>GLN | | 0.69 | DESTABILIZING | 1.05 | 0.31 |
| A:PHE246>GLN | | 0.69 | DESTABILIZING | 0.94 | 0.35 |
| A:GLN284>MET | | 0.7 | DESTABILIZING | 1.69 | -0.14 |
| A:GLN284>THR | | 0.7 | DESTABILIZING | 1.62 | -0.22 |
| A:TRP179>CYS | | 0.72 | DESTABILIZING | 1.96 | -0.53 |
| A:TRP179>PRO | | 0.74 | DESTABILIZING | 2.03 | -0.59 |
| A:TRP179>THR | | 0.75 | DESTABILIZING | 2.06 | -0.59 |
| A:GLU235>ASN | | 0.75 | DESTABILIZING | 2.22 | -0.75 |
| A:GLN284>ASP | | 0.75 | DESTABILIZING | 0.92 | 0.59 |
| A:TRP179>ALA | | 0.76 | DESTABILIZING | 2.07 | -0.59 |
| A:TRP381>THR | | 0.76 | DESTABILIZING | 1.91 | -0.42 |
| A:ASN396>SER | | 0.76 | DESTABILIZING | 1.42 | -0.13 |
| A:LEU314>ALA | | 0.77 | DESTABILIZING | 1.32 | 0.02 |
| A:ASN396>ALA | | 0.77 | DESTABILIZING | 1.66 | -0.16 |
| A:TRP179>GLY | | 0.78 | DESTABILIZING | 2.11 | -0.59 |
| A:PHE246>LEU | | 0.78 | DESTABILIZING | 1.37 | 0.08 |
| A:TRP381>PRO | | 0.78 | DESTABILIZING | 2.07 | -0.55 |
| A:TRP179>SER | | 0.79 | DESTABILIZING | 2.14 | -0.59 |
| A:TRP381>ARG | | 0.82 | DESTABILIZING | 2.19 | -0.54 |
| A:TYR244>MET | | 0.83 | DESTABILIZING | 1.72 | -0.12 |
| A:GLN284>ASN | | 0.84 | DESTABILIZING | 1.9 | -0.17 |
| A:TYR313>ASN | | 0.84 | DESTABILIZING | 1.54 | 0.13 |
| A:TYR313>ILE | | 0.84 | DESTABILIZING | 1.81 | -0.09 |
| A:TRP381>CYS | | 0.84 | DESTABILIZING | 2.14 | -0.47 |
| A:TYR244>HIS | | 0.86 | DESTABILIZING | 1.74 | -0.09 |
| A:TRP381>SER | | 0.86 | DESTABILIZING | 2.15 | -0.46 |
| A:LEU314>GLY | | 0.88 | DESTABILIZING | 1.46 | 0.05 |
| A:TRP381>ALA | | 0.89 | DESTABILIZING | 2.22 | -0.48 |
| A:TRP179>ASP | | 0.92 | DESTABILIZING | 1.84 | -0.01 |
| A:TYR244>GLN | | 0.92 | DESTABILIZING | 1.81 | -0.04 |
| A:PHE246>ILE | | 0.92 | DESTABILIZING | 1.56 | 0.15 |
| A:TYR313>THR | | 0.93 | DESTABILIZING | 1.82 | -0.15 |
| A:GLN284>CYS | | 0.94 | DESTABILIZING | 2.08 | -0.19 |
| A:TRP381>GLY | | 0.94 | DESTABILIZING | 2.31 | -0.48 |
| A:ASN396>GLY | | 0.95 | DESTABILIZING | 2 | -0.19 |
| A:TYR244>LEU | | 0.96 | DESTABILIZING | 2.05 | -0.19 |
| A:PHE246>ARG | | 0.96 | DESTABILIZING | 1.87 | -0.05 |
| A:GLU235>LYS | | 0.97 | DESTABILIZING | 0.35 | 1.55 |
| A:GLN284>SER | | 0.97 | DESTABILIZING | 2.04 | -0.15 |
| A:TRP381>ASP | | 0.98 | DESTABILIZING | 1.72 | 0.23 |
| A:TYR244>GLU | | 1.04 | DESTABILIZING | 1.86 | 0.19 |
| A:GLN284>ARG | | 1.05 | DESTABILIZING | 1.94 | 0.25 |
| A:TRP179>GLU | | 1.06 | DESTABILIZING | 1.07 | 1.04 |
| A:GLU235>VAL | | 1.07 | DESTABILIZING | 2.91 | -0.79 |
| A:TYR244>ILE | | 1.07 | DESTABILIZING | 2.12 | -0.08 |
| A:PHE246>GLU | | 1.07 | DESTABILIZING | 1.09 | 0.95 |
| A:GLN284>ALA | | 1.11 | DESTABILIZING | 2.37 | -0.19 |
| A:PHE246>ASN | | 1.14 | DESTABILIZING | 1.84 | 0.27 |
| A:GLU235>ASP | | 1.17 | DESTABILIZING | 2.38 | -0.08 |
| A:TYR244>VAL | | 1.17 | DESTABILIZING | 2.39 | -0.15 |
| A:TYR313>PRO | | 1.17 | DESTABILIZING | 2.49 | -0.25 |
| A:TYR244>ASN | | 1.19 | DESTABILIZING | 2.44 | -0.15 |
| A:TYR244>THR | | 1.19 | DESTABILIZING | 2.49 | -0.22 |
| A:TYR244>PRO | | 1.21 | DESTABILIZING | 2.48 | -0.17 |
| A:TYR244>ASP | | 1.23 | DESTABILIZING | 2.36 | 0.02 |
| A:GLN284>GLY | | 1.23 | DESTABILIZING | 2.58 | -0.17 |
| A:TYR244>SER | | 1.24 | DESTABILIZING | 2.56 | -0.2 |
| A:PHE246>PRO | | 1.25 | DESTABILIZING | 2.28 | 0.02 |
| A:TYR244>CYS | | 1.26 | DESTABILIZING | 2.64 | -0.22 |
| A:GLN284>TYR | | 1.27 | DESTABILIZING | 3.11 | -0.45 |
| A:GLU235>PRO | | 1.29 | DESTABILIZING | 3.32 | -0.78 |
| A:TYR244>ALA | | 1.3 | DESTABILIZING | 2.71 | -0.24 |
| A:PHE246>THR | | 1.31 | DESTABILIZING | 2.42 | 0 |
| A:PHE246>VAL | | 1.31 | DESTABILIZING | 2.33 | 0.09 |
| A:GLU235>THR | | 1.32 | DESTABILIZING | 3.31 | -0.72 |
| A:TYR244>GLY | | 1.32 | DESTABILIZING | 2.75 | -0.24 |
| A:TYR313>GLU | | 1.34 | DESTABILIZING | -0.22 | 2.8 |
| A:GLU235>CYS | | 1.4 | DESTABILIZING | 3.57 | -0.79 |
| A:PHE246>CYS | | 1.4 | DESTABILIZING | 2.59 | 0.01 |
| A:PHE246>SER | | 1.4 | DESTABILIZING | 2.57 | 0.02 |
| A:GLU235>SER | | 1.42 | DESTABILIZING | 3.44 | -0.68 |
| A:PHE246>ALA | | 1.43 | DESTABILIZING | 2.66 | -0.02 |
| A:TYR313>VAL | | 1.45 | DESTABILIZING | 2.95 | -0.14 |
| A:PHE246>ASP | | 1.46 | DESTABILIZING | 2.06 | 0.68 |
| A:PHE246>GLY | | 1.47 | DESTABILIZING | 2.66 | 0.02 |
| A:GLU235>TRP | | 1.49 | DESTABILIZING | 3.55 | -0.55 |
| A:TYR313>SER | | 1.51 | DESTABILIZING | 2.56 | -0.13 |
| A:TYR313>ASP | | 1.55 | DESTABILIZING | 2.11 | 0.93 |
| A:TYR313>CYS | | 1.59 | DESTABILIZING | 3.27 | -0.16 |
| A:GLU235>ALA | | 1.61 | DESTABILIZING | 4.01 | -0.84 |
| A:TYR313>ALA | | 1.77 | DESTABILIZING | 3.55 | -0.22 |
| A:GLU235>GLY | | 1.81 | DESTABILIZING | 4.42 | -0.88 |
| A:GLN284>TRP | | 1.98 | DESTABILIZING | 4.75 | -0.62 |
| A:TYR313>GLY | | 2.41 | DESTABILIZING | 4.55 | -0.22 |
| Multipoint simultaneous mutagenesis | | | | | |
| A:TYR313>GLY.A:GLU340>ARG.A:ASN396>ARG | | 5.22 | DESTABILIZING | 10.96 | -1.28 |

**Part 5. Results of analysis using the 2V3D PDB structure**

| Mutation | Mutation Energy  (kcal/mol) | | Effect of Mutation | vDW Term | Electrostatic Term |
| --- | --- | --- | --- | --- | --- |
| Alanine scanning | | | | | |
| B:SER345>ALA | | 0.03 | NEUTRAL | 0.13 | -0.03 |
| B:PHE128>ALA | | 0.1 | NEUTRAL | 0.46 | -0.26 |
| B:ALA238>ALA | | 0.11 | NEUTRAL | 0.25 | -0.03 |
| B:ASP127>ALA | | 0.17 | NEUTRAL | 1.38 | -1.05 |
| B:ASN234>ALA | | 0.25 | NEUTRAL | 1.03 | -0.58 |
| B:ASN396>ALA | | 0.49 | NEUTRAL | 1.13 | -0.22 |
| A:PHE316>ALA | | 0.51 | DESTABILIZING | 0.96 | -0.01 |
| A:PHE347>ALA | | 0.59 | DESTABILIZING | 1.2 | -0.01 |
| B:LYS346>ALA | | 0.63 | DESTABILIZING | 1.35 | -0.09 |
| B:TRP381>ALA | | 0.69 | DESTABILIZING | 1.58 | -0.24 |
| B:TRP179>ALA | | 0.75 | DESTABILIZING | 1.91 | -0.44 |
| B:PHE246>ALA | | 0.88 | DESTABILIZING | 1.94 | -0.39 |
| B:LEU314>ALA | | 0.9 | DESTABILIZING | 1.71 | -0.01 |
| B:GLN284>ALA | | 0.92 | DESTABILIZING | 2.14 | -0.36 |
| B:GLU340>ALA | | 0.95 | DESTABILIZING | 1.75 | 0.11 |
| B:GLU235>ALA | | 1.02 | DESTABILIZING | 4.36 | -2.34 |
| B:TYR244>ALA | | 1.63 | DESTABILIZING | 3.55 | -0.36 |
| Saturation mutagenesis | | | | | |
| B:GLU235>LYS | | -0.92 | STABILIZING | -0.23 | -1.68 |
| B:GLU235>TYR | | -0.91 | STABILIZING | 0.09 | -1.86 |
| B:GLU235>GLN | | -0.88 | STABILIZING | -0.03 | -1.75 |
| B:GLU235>PHE | | -0.82 | STABILIZING | 0.49 | -2.08 |
| B:PHE246>LYS | | -0.68 | STABILIZING | -1.12 | -0.34 |
| B:TRP381>MET | | -0.53 | STABILIZING | -1.07 | 0.03 |
| A:PHE316>ARG | | -0.51 | STABILIZING | -1.21 | 0.16 |
| A:PHE347>TYR | | -0.41 | NEUTRAL | -1.01 | 0.1 |
| B:GLU235>LEU | | -0.33 | NEUTRAL | 1.64 | -2.27 |
| B:GLU235>HIS | | -0.31 | NEUTRAL | 1.55 | -2.15 |
| B:GLU235>ILE | | -0.29 | NEUTRAL | 1.63 | -2.21 |
| A:PHE347>TRP | | -0.13 | NEUTRAL | -0.26 | 0 |
| A:PHE316>TRP | | -0.12 | NEUTRAL | -0.3 | 0.05 |
| B:PHE246>TRP | | -0.11 | NEUTRAL | -1.09 | 0.89 |
| B:GLN284>LEU | | -0.08 | NEUTRAL | 0.2 | -0.33 |
| B:TRP381>TYR | | -0.08 | NEUTRAL | 0.11 | -0.27 |
| A:PHE347>PHE | | -0.07 | NEUTRAL | -0.18 | 0 |
| B:TYR244>TYR | | -0.04 | NEUTRAL | 0 | -0.07 |
| B:PHE246>PHE | | -0.04 | NEUTRAL | -0.01 | -0.07 |
| B:GLN284>GLN | | -0.04 | NEUTRAL | -0.03 | -0.04 |
| B:GLU235>GLU | | -0.03 | NEUTRAL | -0.01 | -0.04 |
| B:LYS346>LYS | | -0.03 | NEUTRAL | -0.05 | 0 |
| B:TRP381>TRP | | -0.02 | NEUTRAL | 0 | -0.04 |
| B:TRP179>TRP | | -0.01 | NEUTRAL | 0.01 | -0.03 |
| B:GLU340>GLU | | -0.01 | NEUTRAL | 0 | -0.02 |
| B:TRP381>GLN | | -0.01 | NEUTRAL | 0.04 | -0.09 |
| A:PHE316>PHE | | 0.01 | NEUTRAL | 0.02 | 0 |
| B:LEU314>LEU | | 0.02 | NEUTRAL | 0.03 | 0.01 |
| B:LYS346>HIS | | 0.02 | NEUTRAL | -0.07 | 0 |
| B:LYS346>PHE | | 0.03 | NEUTRAL | 0.25 | -0.08 |
| B:PHE246>MET | | 0.06 | NEUTRAL | 0.44 | -0.39 |
| B:GLU235>ARG | | 0.07 | NEUTRAL | 2.11 | -2 |
| B:PHE246>TYR | | 0.07 | NEUTRAL | -0.75 | 0.9 |
| A:PHE316>LYS | | 0.08 | NEUTRAL | -0.01 | 0.13 |
| B:LEU314>ASN | | 0.08 | NEUTRAL | -0.11 | 0.21 |
| B:GLU340>GLN | | 0.08 | NEUTRAL | -0.19 | 0.34 |
| B:TYR244>TRP | | 0.1 | NEUTRAL | 0.33 | -0.14 |
| B:LEU314>PHE | | 0.11 | NEUTRAL | 0.29 | -0.04 |
| A:PHE347>LYS | | 0.13 | NEUTRAL | -0.34 | 0.24 |
| B:TRP381>PHE | | 0.13 | NEUTRAL | 0.56 | -0.3 |
| B:LYS346>TRP | | 0.15 | NEUTRAL | 0.47 | -0.06 |
| B:LEU314>ILE | | 0.16 | NEUTRAL | 0.27 | 0.03 |
| B:TRP381>ILE | | 0.18 | NEUTRAL | 0.54 | -0.2 |
| A:PHE316>HIS | | 0.19 | NEUTRAL | 0.38 | -0.02 |
| A:PHE316>MET | | 0.19 | NEUTRAL | 0.35 | -0.01 |
| B:TRP381>LEU | | 0.19 | NEUTRAL | 0.61 | -0.24 |
| B:TYR244>ARG | | 0.2 | NEUTRAL | 0.3 | 0.08 |
| B:PHE246>HIS | | 0.2 | NEUTRAL | 0.58 | -0.25 |
| B:LYS346>LEU | | 0.2 | NEUTRAL | 0.52 | -0.09 |
| B:LEU314>TRP | | 0.22 | NEUTRAL | 0.46 | 0.01 |
| B:LYS346>TYR | | 0.24 | NEUTRAL | 0.62 | -0.05 |
| A:PHE347>MET | | 0.25 | NEUTRAL | 0.37 | -0.03 |
| B:GLU235>MET | | 0.26 | NEUTRAL | 2.82 | -2.29 |
| A:PHE316>GLN | | 0.3 | NEUTRAL | 0.45 | 0.1 |
| A:PHE316>TYR | | 0.3 | NEUTRAL | 0.45 | 0.16 |
| B:TRP179>TYR | | 0.3 | NEUTRAL | 0.55 | 0.05 |
| B:GLU340>TYR | | 0.31 | NEUTRAL | 0.54 | 0.05 |
| B:GLN284>ILE | | 0.32 | NEUTRAL | 0.93 | -0.29 |
| B:LYS346>MET | | 0.32 | NEUTRAL | 0.78 | -0.09 |
| B:TRP179>PHE | | 0.33 | NEUTRAL | 0.88 | -0.23 |
| B:PHE246>LEU | | 0.33 | NEUTRAL | 0.84 | -0.27 |
| B:GLN284>ASN | | 0.35 | NEUTRAL | 0.73 | -0.02 |
| B:LEU314>GLN | | 0.35 | NEUTRAL | 0.37 | 0.17 |
| A:PHE316>GLU | | 0.36 | NEUTRAL | 0.48 | 0.2 |
| A:PHE347>HIS | | 0.36 | NEUTRAL | 0.5 | 0.02 |
| A:PHE316>ILE | | 0.37 | NEUTRAL | 0.72 | -0.01 |
| A:PHE316>LEU | | 0.37 | NEUTRAL | 0.74 | -0.03 |
| B:TRP179>HIS | | 0.38 | NEUTRAL | 1.11 | -0.36 |
| B:LYS346>ILE | | 0.39 | NEUTRAL | 0.86 | -0.11 |
| B:GLN284>LYS | | 0.4 | NEUTRAL | 1.29 | -0.5 |
| B:LYS346>ARG | | 0.4 | NEUTRAL | 0.84 | -0.08 |
| B:PHE246>ILE | | 0.41 | NEUTRAL | 1.07 | -0.34 |
| A:PHE347>ARG | | 0.42 | NEUTRAL | 0.44 | 0.12 |
| A:PHE347>LEU | | 0.42 | NEUTRAL | 0.71 | -0.02 |
| B:TRP381>VAL | | 0.42 | NEUTRAL | 1.02 | -0.19 |
| A:PHE316>ASN | | 0.43 | NEUTRAL | 0.81 | 0.02 |
| B:LYS346>GLU | | 0.43 | NEUTRAL | 0.76 | 0.06 |
| B:TRP381>ASN | | 0.43 | NEUTRAL | 0.96 | -0.12 |
| B:LYS346>PRO | | 0.44 | NEUTRAL | 1.09 | -0.15 |
| B:LYS346>VAL | | 0.44 | NEUTRAL | 0.98 | -0.1 |
| B:TRP179>LYS | | 0.45 | NEUTRAL | 1.84 | -0.94 |
| B:LEU314>VAL | | 0.45 | NEUTRAL | 0.81 | 0.01 |
| B:GLU340>ILE | | 0.45 | NEUTRAL | 0.7 | 0.18 |
| A:PHE316>PRO | | 0.46 | NEUTRAL | 0.91 | -0.02 |
| A:PHE316>VAL | | 0.46 | NEUTRAL | 0.89 | -0.01 |
| B:LEU314>ASP | | 0.46 | NEUTRAL | 0.12 | 0.76 |
| A:PHE316>ASP | | 0.47 | NEUTRAL | 0.81 | 0.1 |
| A:PHE347>ILE | | 0.47 | NEUTRAL | 0.97 | -0.01 |
| A:PHE347>PRO | | 0.47 | NEUTRAL | 0.95 | 0 |
| B:TRP179>LEU | | 0.47 | NEUTRAL | 1.27 | -0.34 |
| B:GLU235>VAL | | 0.47 | NEUTRAL | 3.26 | -2.31 |
| A:PHE316>THR | | 0.48 | NEUTRAL | 0.94 | -0.01 |
| B:LEU314>GLU | | 0.48 | NEUTRAL | 0.2 | 0.64 |
| B:GLU340>LEU | | 0.48 | NEUTRAL | 0.85 | 0.1 |
| A:PHE316>CYS | | 0.49 | NEUTRAL | 0.95 | -0.01 |
| A:PHE316>SER | | 0.49 | NEUTRAL | 0.94 | 0 |
| B:TRP381>LYS | | 0.49 | NEUTRAL | 1.19 | -0.22 |
| B:GLN284>PRO | | 0.5 | NEUTRAL | 1.4 | -0.42 |
| B:GLU340>ARG | | 0.5 | NEUTRAL | 0.08 | 0.93 |
| B:LYS346>GLN | | 0.5 | NEUTRAL | 0.99 | -0.09 |
| A:PHE316>ALA | | 0.51 | DESTABILIZING | 0.96 | -0.01 |
| A:PHE347>VAL | | 0.51 | DESTABILIZING | 1.04 | 0 |
| B:TRP179>MET | | 0.51 | DESTABILIZING | 1.57 | -0.55 |
| B:TRP381>GLU | | 0.51 | DESTABILIZING | 0.17 | 0.81 |
| A:PHE316>GLY | | 0.52 | DESTABILIZING | 0.97 | -0.02 |
| A:PHE347>ASN | | 0.52 | DESTABILIZING | 0.91 | 0.01 |
| B:LYS346>ASP | | 0.52 | DESTABILIZING | 0.86 | 0.17 |
| B:LYS346>THR | | 0.52 | DESTABILIZING | 1.14 | -0.11 |
| B:TRP381>HIS | | 0.52 | DESTABILIZING | 1.33 | -0.3 |
| B:TRP381>THR | | 0.53 | DESTABILIZING | 1.22 | -0.19 |
| A:PHE347>GLN | | 0.54 | DESTABILIZING | 0.56 | 0.04 |
| A:PHE347>THR | | 0.54 | DESTABILIZING | 1.08 | 0.01 |
| A:PHE347>ASP | | 0.56 | DESTABILIZING | 0.9 | 0.13 |
| A:PHE347>GLU | | 0.56 | DESTABILIZING | 0.66 | 0.2 |
| B:LYS346>CYS | | 0.56 | DESTABILIZING | 1.28 | -0.09 |
| A:PHE347>CYS | | 0.57 | DESTABILIZING | 1.15 | 0 |
| A:PHE347>SER | | 0.57 | DESTABILIZING | 1.17 | -0.01 |
| B:TRP179>ILE | | 0.57 | DESTABILIZING | 1.52 | -0.4 |
| B:GLN284>VAL | | 0.57 | DESTABILIZING | 1.45 | -0.32 |
| B:LEU314>ARG | | 0.57 | DESTABILIZING | 0.45 | 0.5 |
| B:LEU314>THR | | 0.57 | DESTABILIZING | 1.02 | 0.02 |
| A:PHE347>ALA | | 0.59 | DESTABILIZING | 1.2 | -0.01 |
| B:GLU235>ASN | | 0.59 | DESTABILIZING | 3.13 | -1.97 |
| B:TYR244>PHE | | 0.59 | DESTABILIZING | 1.56 | -0.37 |
| B:LYS346>ASN | | 0.59 | DESTABILIZING | 1.28 | -0.11 |
| B:TRP179>GLN | | 0.6 | DESTABILIZING | 1.14 | 0.05 |
| B:GLN284>THR | | 0.6 | DESTABILIZING | 1.53 | -0.35 |
| B:GLU340>ASP | | 0.6 | DESTABILIZING | 0.94 | 0.25 |
| B:TRP381>CYS | | 0.6 | DESTABILIZING | 1.45 | -0.27 |
| B:LEU314>CYS | | 0.61 | DESTABILIZING | 1.13 | 0.08 |
| B:TRP381>ASP | | 0.61 | DESTABILIZING | 1 | 0.21 |
| A:PHE347>GLY | | 0.63 | DESTABILIZING | 1.28 | -0.02 |
| B:LYS346>ALA | | 0.63 | DESTABILIZING | 1.35 | -0.09 |
| B:LYS346>SER | | 0.63 | DESTABILIZING | 1.27 | -0.08 |
| B:TRP381>SER | | 0.63 | DESTABILIZING | 1.45 | -0.24 |
| B:LEU314>PRO | | 0.64 | DESTABILIZING | 1.2 | -0.01 |
| B:TRP381>PRO | | 0.64 | DESTABILIZING | 1.54 | -0.31 |
| B:GLN284>GLU | | 0.65 | DESTABILIZING | 0.17 | 1.13 |
| B:TRP179>ASN | | 0.67 | DESTABILIZING | 1.58 | -0.26 |
| B:TRP179>THR | | 0.67 | DESTABILIZING | 1.78 | -0.46 |
| B:GLU235>THR | | 0.67 | DESTABILIZING | 3.58 | -2.28 |
| B:GLU340>ASN | | 0.67 | DESTABILIZING | 1.08 | 0.25 |
| B:LYS346>GLY | | 0.67 | DESTABILIZING | 1.43 | -0.08 |
| B:TRP179>VAL | | 0.68 | DESTABILIZING | 1.76 | -0.41 |
| B:LEU314>SER | | 0.68 | DESTABILIZING | 1.05 | 0.13 |
| B:PHE246>GLU | | 0.69 | DESTABILIZING | 0.56 | 0.74 |
| B:TRP381>ALA | | 0.69 | DESTABILIZING | 1.58 | -0.24 |
| B:TRP179>PRO | | 0.72 | DESTABILIZING | 1.88 | -0.47 |
| B:GLU235>PRO | | 0.72 | DESTABILIZING | 3.81 | -2.38 |
| B:PHE246>GLN | | 0.72 | DESTABILIZING | 1.33 | 0.01 |
| B:TRP179>CYS | | 0.73 | DESTABILIZING | 1.88 | -0.44 |
| B:PHE246>VAL | | 0.73 | DESTABILIZING | 1.67 | -0.37 |
| B:GLU340>VAL | | 0.73 | DESTABILIZING | 1.25 | 0.17 |
| B:TRP179>ALA | | 0.75 | DESTABILIZING | 1.91 | -0.44 |
| B:TRP179>SER | | 0.75 | DESTABILIZING | 1.89 | -0.43 |
| B:PHE246>PRO | | 0.75 | DESTABILIZING | 1.72 | -0.39 |
| B:LEU314>TYR | | 0.75 | DESTABILIZING | 1.43 | -0.08 |
| B:PHE246>ASN | | 0.76 | DESTABILIZING | 1.46 | -0.08 |
| B:GLU340>THR | | 0.77 | DESTABILIZING | 1.35 | 0.16 |
| B:TRP381>GLY | | 0.77 | DESTABILIZING | 1.71 | -0.23 |
| B:GLN284>CYS | | 0.78 | DESTABILIZING | 1.9 | -0.33 |
| B:LEU314>MET | | 0.78 | DESTABILIZING | 1.46 | -0.04 |
| B:GLN284>ASP | | 0.79 | DESTABILIZING | 0.93 | 0.65 |
| B:TRP179>GLY | | 0.8 | DESTABILIZING | 1.98 | -0.42 |
| B:PHE246>CYS | | 0.82 | DESTABILIZING | 1.84 | -0.36 |
| B:PHE246>THR | | 0.82 | DESTABILIZING | 1.83 | -0.36 |
| B:GLU340>PRO | | 0.83 | DESTABILIZING | 1.48 | 0.15 |
| B:PHE246>SER | | 0.85 | DESTABILIZING | 1.85 | -0.35 |
| B:GLU340>CYS | | 0.85 | DESTABILIZING | 1.63 | 0.05 |
| B:PHE246>ARG | | 0.86 | DESTABILIZING | 1.84 | -0.34 |
| B:GLN284>ARG | | 0.86 | DESTABILIZING | 1.79 | -0.19 |
| B:PHE246>ALA | | 0.88 | DESTABILIZING | 1.94 | -0.39 |
| B:GLU340>HIS | | 0.89 | DESTABILIZING | 1.94 | -0.15 |
| B:GLN284>SER | | 0.9 | DESTABILIZING | 2.1 | -0.39 |
| B:LEU314>ALA | | 0.9 | DESTABILIZING | 1.71 | -0.01 |
| B:GLU340>SER | | 0.9 | DESTABILIZING | 1.61 | 0.16 |
| B:GLU235>CYS | | 0.91 | DESTABILIZING | 4.19 | -2.37 |
| B:PHE246>GLY | | 0.91 | DESTABILIZING | 2 | -0.4 |
| B:GLN284>ALA | | 0.92 | DESTABILIZING | 2.14 | -0.36 |
| B:GLU235>SER | | 0.93 | DESTABILIZING | 4.07 | -2.28 |
| B:TRP179>GLU | | 0.94 | DESTABILIZING | 1.16 | 0.71 |
| B:TRP179>ASP | | 0.95 | DESTABILIZING | 1.74 | 0.14 |
| B:LEU314>LYS | | 0.95 | DESTABILIZING | 1.26 | 0.45 |
| B:GLU340>ALA | | 0.95 | DESTABILIZING | 1.75 | 0.11 |
| B:GLU340>PHE | | 0.96 | DESTABILIZING | 1.42 | 0.54 |
| B:GLN284>HIS | | 0.98 | DESTABILIZING | 2.03 | -0.04 |
| B:LEU314>GLY | | 0.98 | DESTABILIZING | 1.88 | -0.01 |
| B:LEU314>HIS | | 0.98 | DESTABILIZING | 2.09 | -0.2 |
| B:PHE246>ASP | | 0.99 | DESTABILIZING | 1.44 | 0.39 |
| B:GLU340>GLY | | 1.01 | DESTABILIZING | 1.9 | 0.09 |
| B:GLU235>ALA | | 1.02 | DESTABILIZING | 4.36 | -2.34 |
| B:GLN284>GLY | | 1.02 | DESTABILIZING | 2.29 | -0.37 |
| B:TRP179>ARG | | 1.06 | DESTABILIZING | 2.56 | -0.43 |
| B:TYR244>LYS | | 1.08 | DESTABILIZING | 2.63 | -0.51 |
| B:TYR244>GLN | | 1.09 | DESTABILIZING | 2.29 | -0.18 |
| B:TYR244>MET | | 1.09 | DESTABILIZING | 2.53 | -0.38 |
| B:TYR244>HIS | | 1.22 | DESTABILIZING | 2.77 | -0.36 |
| B:TYR244>LEU | | 1.23 | DESTABILIZING | 2.79 | -0.37 |
| B:GLU235>GLY | | 1.24 | DESTABILIZING | 4.83 | -2.39 |
| B:GLU235>TRP | | 1.36 | DESTABILIZING | 3.39 | -0.63 |
| B:TYR244>GLU | | 1.36 | DESTABILIZING | 2.39 | 0.26 |
| B:GLU340>TRP | | 1.36 | DESTABILIZING | 2.35 | 0.39 |
| B:TYR244>ILE | | 1.42 | DESTABILIZING | 3.17 | -0.37 |
| B:GLN284>MET | | 1.5 | DESTABILIZING | 3.58 | -0.58 |
| B:TYR244>ASN | | 1.51 | DESTABILIZING | 3.26 | -0.28 |
| B:TYR244>VAL | | 1.53 | DESTABILIZING | 3.37 | -0.35 |
| B:TYR244>THR | | 1.56 | DESTABILIZING | 3.44 | -0.37 |
| B:TYR244>PRO | | 1.58 | DESTABILIZING | 3.49 | -0.38 |
| B:TYR244>CYS | | 1.6 | DESTABILIZING | 3.54 | -0.38 |
| B:TYR244>SER | | 1.6 | DESTABILIZING | 3.52 | -0.36 |
| B:TYR244>ALA | | 1.63 | DESTABILIZING | 3.55 | -0.36 |
| B:TYR244>ASP | | 1.63 | DESTABILIZING | 3.25 | -0.03 |
| B:TYR244>GLY | | 1.69 | DESTABILIZING | 3.67 | -0.36 |
| B:GLU340>MET | | 1.73 | DESTABILIZING | 3.5 | -0.04 |
| B:GLN284>TYR | | 1.85 | DESTABILIZING | 3.92 | -0.19 |
| B:TRP381>ARG | | 1.93 | DESTABILIZING | 4.25 | -0.4 |
| B:GLU235>ASP | | 2.01 | DESTABILIZING | 5.14 | -1.14 |
| B:GLU340>LYS | | 2.02 | DESTABILIZING | 3.15 | 0.89 |
| B:GLN284>PHE | | 2.22 | DESTABILIZING | 4.99 | -0.49 |
| B:GLN284>TRP | | 2.22 | DESTABILIZING | 5.07 | -0.59 |
| Multipoint simultaneous mutagenesis | | | | | |
| B:TYR313>GLY.B:GLU340>ARG.B:ASN396>ARG | | 4 | DESTABILIZING | 8.34 | -0.59 |

**Part 6. Results of analysis using the 2XWE PDB structure**

| Mutation | Mutation Energy  (kcal/mol) | | Effect of Mutation | vDW Term | Electrostatic Term |
| --- | --- | --- | --- | --- | --- |
| Alanine scanning | | | | | |
| B:ASP127>ALA | -0.42 | NEUTRAL | | 0.88 | -1.73 |
| B:SER345>ALA | -0.07 | NEUTRAL | | -0.09 | 0.22 |
| B:ASN234>ALA | 0.15 | NEUTRAL | | 0.83 | -0.58 |
| B:CYS342>ALA | 0.15 | NEUTRAL | | 0.28 | -0.05 |
| B:GLU235>ALA | 0.21 | NEUTRAL | | 2.13 | -1.74 |
| B:PHE128>ALA | 0.28 | NEUTRAL | | 0.84 | -0.3 |
| B:PRO245>ALA | 0.3 | NEUTRAL | | 0.52 | 0.04 |
| B:GLN284>ALA | 0.48 | NEUTRAL | | 1.08 | -0.19 |
| B:TRP179>ALA | 0.5 | NEUTRAL | | 1.66 | -0.69 |
| B:TYR313>ALA | 0.51 | DESTABILIZING | | 0.75 | -0.14 |
| A:TRP348>ALA | 0.55 | DESTABILIZING | | 1.2 | 0.02 |
| B:VAL398>ALA | 0.55 | DESTABILIZING | | 0.95 | 0.01 |
| B:GLU340>ALA | 0.84 | DESTABILIZING | | 2.09 | -0.44 |
| B:ASN396>ALA | 0.92 | DESTABILIZING | | 2.25 | -0.24 |
| B:TRP381>ALA | 1.33 | DESTABILIZING | | 2.94 | -0.35 |
| B:PHE246>ALA | 1.65 | DESTABILIZING | | 2.87 | -0.02 |
| B:TYR244>ALA | 1.66 | DESTABILIZING | | 3.56 | -0.33 |
| Saturation mutagenesis | | | | | |
| B:TYR313>ILE | -0.51 | STABILIZING | | -1.11 | 0.02 |
| B:ASN396>ILE | -0.36 | NEUTRAL | | -0.37 | -0.28 |
| B:ASN396>HIS | -0.33 | NEUTRAL | | -0.24 | -0.38 |
| B:ASN396>LEU | -0.32 | NEUTRAL | | -0.31 | -0.19 |
| B:VAL398>GLN | -0.32 | NEUTRAL | | -1.45 | 0.98 |
| B:TYR313>PRO | -0.27 | NEUTRAL | | -0.84 | -0.13 |
| B:ASN396>GLN | -0.16 | NEUTRAL | | -0.32 | -0.03 |
| B:VAL398>ILE | -0.11 | NEUTRAL | | -0.44 | 0.31 |
| B:TYR313>ARG | -0.09 | NEUTRAL | | -0.71 | 0.35 |
| B:ASN396>THR | -0.07 | NEUTRAL | | 0.17 | -0.4 |
| B:TYR313>THR | -0.04 | NEUTRAL | | -0.63 | -0.09 |
| B:TRP348>ARG | 0.01 | NEUTRAL | | -0.01 | 0.02 |
| B:TRP348>HIS | 0.01 | NEUTRAL | | 0.03 | 0 |
| B:TRP348>ILE | 0.02 | NEUTRAL | | 0.03 | 0 |
| B:TRP348>MET | 0.02 | NEUTRAL | | 0.02 | 0 |
| B:TRP348>PRO | 0.02 | NEUTRAL | | 0.03 | 0.01 |
| B:TRP348>ALA | 0.03 | NEUTRAL | | 0.05 | 0.01 |
| B:TRP348>ASP | 0.03 | NEUTRAL | | 0.03 | -0.01 |
| B:TRP348>CYS | 0.03 | NEUTRAL | | 0.04 | 0.01 |
| B:TRP348>GLN | 0.03 | NEUTRAL | | 0.03 | 0.01 |
| B:TRP348>GLY | 0.03 | NEUTRAL | | 0.05 | 0.01 |
| B:TRP348>LEU | 0.03 | NEUTRAL | | 0.02 | 0 |
| B:TRP348>LYS | 0.03 | NEUTRAL | | 0.01 | 0.04 |
| B:TRP348>SER | 0.04 | NEUTRAL | | 0.04 | 0 |
| B:TRP348>THR | 0.04 | NEUTRAL | | 0.03 | 0.01 |
| B:GLU340>GLN | 0.05 | NEUTRAL | | -0.19 | 0.28 |
| B:VAL398>THR | 0.05 | NEUTRAL | | 0.04 | 0.06 |
| B:TYR313>MET | 0.11 | NEUTRAL | | -0.11 | 0.13 |
| B:ASN396>ASP | 0.18 | NEUTRAL | | -0.67 | 1.02 |
| B:VAL398>PRO | 0.22 | NEUTRAL | | 0.29 | 0 |
| B:VAL398>CYS | 0.27 | NEUTRAL | | 0.55 | 0.02 |
| B:VAL398>SER | 0.29 | NEUTRAL | | 0.52 | 0.01 |
| B:VAL398>LYS | 0.3 | NEUTRAL | | 0.45 | 0.11 |
| B:TYR244>ARG | 0.32 | NEUTRAL | | 0.08 | 0.52 |
| B:TYR313>CYS | 0.33 | NEUTRAL | | 0.65 | -0.11 |
| B:PHE246>GLN | 0.35 | NEUTRAL | | 0.25 | 0.37 |
| B:GLU340>LYS | 0.35 | NEUTRAL | | 0.65 | 0.02 |
| B:VAL398>MET | 0.35 | NEUTRAL | | 0.62 | 0.31 |
| B:PHE246>HIS | 0.36 | NEUTRAL | | 0.51 | 0.13 |
| B:GLU340>LEU | 0.39 | NEUTRAL | | 1.15 | -0.39 |
| B:GLU340>ILE | 0.4 | NEUTRAL | | 1.13 | -0.34 |
| B:TYR313>LEU | 0.44 | NEUTRAL | | 0.89 | 0.03 |
| B:ASN396>MET | 0.46 | NEUTRAL | | 1.14 | -0.19 |
| B:TRP381>GLN | 0.47 | NEUTRAL | | 0.49 | 0.4 |
| B:ASN396>SER | 0.47 | NEUTRAL | | 1.24 | -0.39 |
| B:TYR313>ASP | 0.49 | NEUTRAL | | -0.02 | 0.92 |
| B:TYR313>SER | 0.5 | NEUTRAL | | 0.06 | -0.03 |
| B:TYR313>ALA | 0.51 | DESTABILIZING | | 0.75 | -0.14 |
| B:ASN396>CYS | 0.51 | DESTABILIZING | | 1.52 | -0.33 |
| B:VAL398>HIS | 0.52 | DESTABILIZING | | 0.75 | 0.48 |
| B:TYR244>LYS | 0.53 | DESTABILIZING | | 0.99 | 0.01 |
| B:PHE246>MET | 0.53 | DESTABILIZING | | 1.13 | -0.12 |
| B:GLU340>THR | 0.53 | DESTABILIZING | | 1.45 | -0.43 |
| B:VAL398>ALA | 0.55 | DESTABILIZING | | 0.95 | 0.01 |
| B:TRP381>HIS | 0.57 | DESTABILIZING | | 1.45 | -0.3 |
| B:TYR313>HIS | 0.59 | DESTABILIZING | | 1.13 | -0.04 |
| B:GLU340>SER | 0.61 | DESTABILIZING | | 1.65 | -0.47 |
| B:PHE246>LEU | 0.63 | DESTABILIZING | | 1.13 | -0.01 |
| B:TRP381>ILE | 0.66 | DESTABILIZING | | 1.48 | -0.18 |
| B:GLU340>ASP | 0.68 | DESTABILIZING | | 1.41 | -0.07 |
| B:VAL398>GLY | 0.68 | DESTABILIZING | | 1.21 | -0.08 |
| B:TRP381>LEU | 0.69 | DESTABILIZING | | 1.61 | -0.25 |
| B:ASN396>PRO | 0.7 | DESTABILIZING | | 1.62 | -0.07 |
| B:GLU340>PRO | 0.71 | DESTABILIZING | | 1.74 | -0.37 |
| B:GLU340>CYS | 0.77 | DESTABILIZING | | 1.97 | -0.45 |
| B:GLU340>ALA | 0.84 | DESTABILIZING | | 2.09 | -0.44 |
| B:VAL398>LEU | 0.85 | DESTABILIZING | | 0.99 | 0.73 |
| B:PHE246>ILE | 0.89 | DESTABILIZING | | 1.58 | 0.05 |
| B:PHE246>LYS | 0.89 | DESTABILIZING | | 1.26 | 0.4 |
| B:GLU340>GLY | 0.91 | DESTABILIZING | | 2.23 | -0.46 |
| B:ASN396>ALA | 0.92 | DESTABILIZING | | 2.25 | -0.24 |
| B:VAL398>ASP | 0.92 | DESTABILIZING | | 0.22 | 1.47 |
| B:GLU340>HIS | 0.93 | DESTABILIZING | | 2.65 | -0.75 |
| B:TYR313>GLY | 0.94 | DESTABILIZING | | 1.43 | -0.14 |
| B:TRP381>MET | 0.95 | DESTABILIZING | | 2.01 | -0.12 |
| B:TYR244>HIS | 0.98 | DESTABILIZING | | 2 | -0.08 |
| B:TRP381>THR | 1.1 | DESTABILIZING | | 2.44 | -0.28 |
| B:ASN396>GLY | 1.12 | DESTABILIZING | | 2.68 | -0.29 |
| B:TYR244>GLN | 1.18 | DESTABILIZING | | 2.46 | -0.21 |
| B:TYR244>MET | 1.18 | DESTABILIZING | | 2.63 | -0.4 |
| B:TYR244>LEU | 1.2 | DESTABILIZING | | 2.61 | -0.28 |
| B:TRP381>CYS | 1.24 | DESTABILIZING | | 2.8 | -0.33 |
| B:TRP381>SER | 1.25 | DESTABILIZING | | 2.76 | -0.31 |
| B:TRP381>PRO | 1.27 | DESTABILIZING | | 2.85 | -0.37 |
| B:PHE246>PRO | 1.31 | DESTABILIZING | | 2.46 | -0.18 |
| B:TRP381>ALA | 1.33 | DESTABILIZING | | 2.94 | -0.35 |
| B:TRP381>ASP | 1.36 | DESTABILIZING | | 2.19 | 0.51 |
| B:TYR244>ILE | 1.38 | DESTABILIZING | | 2.94 | -0.26 |
| B:TRP381>GLY | 1.39 | DESTABILIZING | | 3.06 | -0.37 |
| B:PHE246>THR | 1.43 | DESTABILIZING | | 2.58 | -0.09 |
| B:PHE246>CYS | 1.51 | DESTABILIZING | | 2.77 | -0.07 |
| B:TYR244>ASP | 1.52 | DESTABILIZING | | 2.88 | 0.12 |
| B:TYR244>PRO | 1.56 | DESTABILIZING | | 3.37 | -0.34 |
| B:TYR244>THR | 1.56 | DESTABILIZING | | 3.36 | -0.31 |
| B:PHE246>ASP | 1.56 | DESTABILIZING | | 2.02 | 0.87 |
| B:TYR244>SER | 1.61 | DESTABILIZING | | 3.48 | -0.36 |
| B:TYR244>CYS | 1.63 | DESTABILIZING | | 3.47 | -0.29 |
| B:PHE246>ALA | 1.65 | DESTABILIZING | | 2.87 | -0.02 |
| B:TYR244>ALA | 1.66 | DESTABILIZING | | 3.56 | -0.33 |
| B:PHE246>GLY | 1.66 | DESTABILIZING | | 2.97 | -0.13 |
| B:PHE246>SER | 1.66 | DESTABILIZING | | 2.96 | -0.11 |
| B:TYR313>LYS | 1.66 | DESTABILIZING | | 2.11 | 0.82 |
| B:ASN396>LYS | 1.69 | DESTABILIZING | | 2.06 | 0.79 |
| B:TYR244>GLY | 1.71 | DESTABILIZING | | 3.65 | -0.33 |
| B:GLU340>MET | 1.76 | DESTABILIZING | | 3.89 | -0.37 |
| B:PHE246>ARG | 1.8 | DESTABILIZING | | 3.55 | -0.05 |
| B:TYR313>GLN | 1.92 | DESTABILIZING | | 2.1 | 0.37 |
| B:TRP381>LYS | 2.46 | DESTABILIZING | | 4.19 | 0.72 |
| B:TRP381>ARG | 2.59 | DESTABILIZING | | 5.56 | -0.42 |
| B:VAL398>ARG | 2.92 | DESTABILIZING | | 5.4 | 0.46 |
| B:ASN396>ARG | 3.35 | DESTABILIZING | | 6.05 | 0.11 |
| B:GLU340>ARG | 3.92 | DESTABILIZING | | 8.54 | -0.69 |
| Multipoint simultaneous mutagenesis | | | | | |
| B:TYR313>GLY.B:GLU340>ARG.B:ASN396>ARG | 3.59 | DESTABILIZING | | 6.28 | -0.31 |


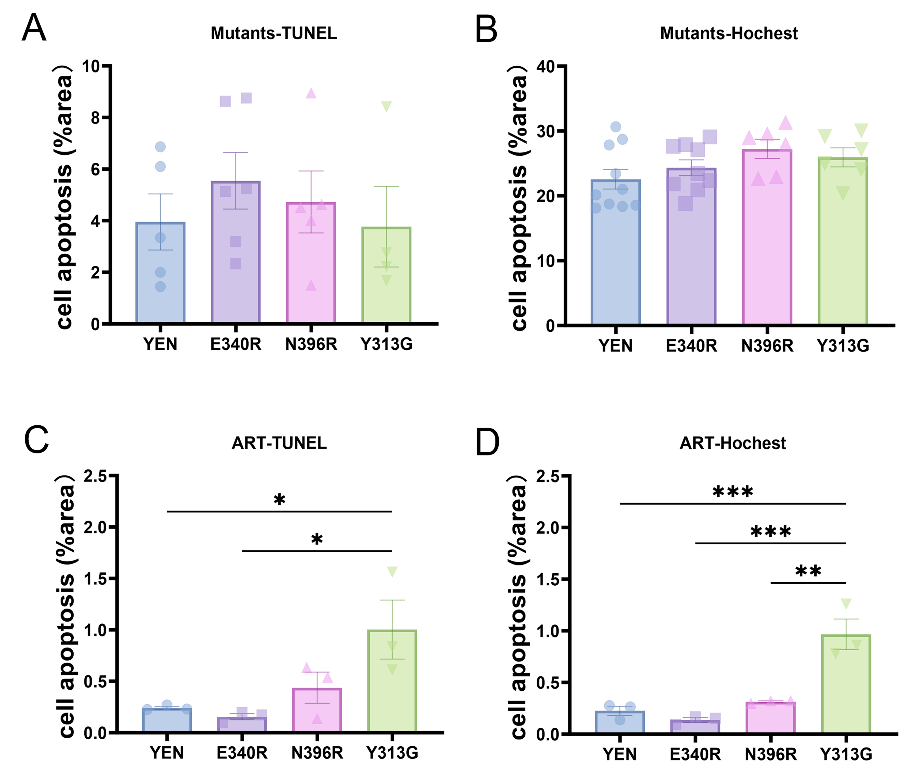
Figure S1. Quantitative analysis of apoptosis in HepG2 cells expressing GBA point mutants (Y313G, E340R, N396R, Y313G/E340R/N396R) in response to artesunate treatment (n=3). **(A, B)** Baseline apoptosis rates without artesunate treatment. **(C, D)** Apoptosis rates after artesunate treatment.
