# Supplementary material for: Artesunate directly targets glucosylceramidase to suppress hepatocellular carcinoma proliferation and trigger apoptosis
Source: Genes Dis. 2026 Jan 20;13(5):102045. doi: 10.1016/j.gendis.2026.102045 (PMC13265689; doi:10.1016/j.gendis.2026.102045)
Supplement: Multimedia component 2 [file mmc2.docx]

**Copyright Transfer Agreement**

The author confirms:

that the work described has not been published before (except in the form of an abstract or as part of a published lecture, review, or thesis);

that it is not under consideration for publication elsewhere;

that its publication has been approved by all co-authors, if any;

that its publication has been approved (tacitly or explicitly) by the responsible authorities at the institution where the work carried out.

The author agrees to publication in the Journal indicated below and also to publication of the article in English by Genes & Diseases in Elsevier's corresponding English-language journal.

The copyright to the English-language article is transferred to Elsevier effective if and when the article is accepted for publication. The author warrants that his/her contribution is original and that he/she has full power to make this grant. The author signs for and accepts responsibility for releasing this material on behalf of any and all co-authors. The copyright transfer covers the exclusive right to reproduce and distribute to article, including reprints, translations, photographic reproductions, microform, electronic form (offline, online) or any other reproductions of similar nature.

Journal: Genes & Diseases

Title of article: Artesunate Directly Targets Glucosylceramidase to Suppress Hepatocellular Carcinoma Proliferation and Trigger Apoptosis
